# Prevalence of c-shaped canal morphology in premolar and molar teeth assessed by cone-beam computed tomography: systematic review and meta-analysis

**DOI:** 10.1186/s12903-025-06946-8

**Published:** 2025-10-22

**Authors:** Faezeh Yousefi, Younes Mohammadi, Elham Shokri

**Affiliations:** 1 Oral and maxillofacial radiology department, Dental research center, Hamadan University of Medical Sciences, Hamadan, Iran; 2 Epidemiology Department, School of Public Health, Hamadan University of Medical Sciences, Hamadan, Iran; 3 Oral and Maxillofacial Radiology, Hamadan University of Medical Sciences, Hamadan, Iran

**Keywords:** Cone-Beam computed tomography, Prevalence, Morphology, Root canal, Systematic review

## Abstract

**Purpose:**

This systematic review aimed to investigate the prevalence of c-shaped canal in mandibular and maxillary premolars and molars using cone-beam computed tomography (CBCT) and the effect of factors (e.g., gender, geographic region and position in the jaw) on the prevalence of this anomaly.

**Materials and methods:**

A search was conducted in 3 databases (PubMed, Scopus and web of science) and studies were selected based on inclusion and exclusion criteria. The inclusion criteria were studies that investigated the prevalence of C-shaped canal using CBCT images until 2024. The I2 statistic was performed to determine heterogeneity and meta-analysis was conducted.

**Results:**

Of 5548 studies, 101 studies were selected for meta-analysis based on inclusion criteria. The highest prevalence of c-shaped canal was in the mandibular second molar (17.3%) and the lowest prevalence was in the maxillary first molar (0.8%). The prevalence of c-shaped canal in the mandibular second molar was higher in women (Female: 23.6%, Male: 16.7%). C-shaped canal in the mandibular second molar was more common in Asian continent compared to other continents. The prevalence of C-shaped canals did not differ significantly in the mandibular second molar on the right and left sides (Right: 21.2%, Left: 23.1%).

**Conclusion:**

Based on the results of this systematic review, the highest prevalence of c-shaped canal was in the mandibular second molar and it was more in Asian continent. Clinicians can consider the possibility of a C-shaped canal on the opposite side if they encounter the presence of a C-shaped canal during root canal therapy on one side. CBCT can be used to obtain sufficient information about the morphology of the root canal to improve the success of the root canal treatment.

**Supplementary Information:**

The online version contains supplementary material available at 10.1186/s12903-025-06946-8.

## Introduction

Root canal anatomy is a complex structure influenced by heredity and ethnic diversity [1]. Anatomical features of the root canal (e.g., an additional canal or root, a C-shaped canal, a calcified canal, and a severely curved root) can affect root canal treatment and pose challenges to the clinician such as missed canals, perforation, root canal transportation [2]. A thorough understanding of root canal morphology is essential for successful root canal treatment.

**Fig. 1:**
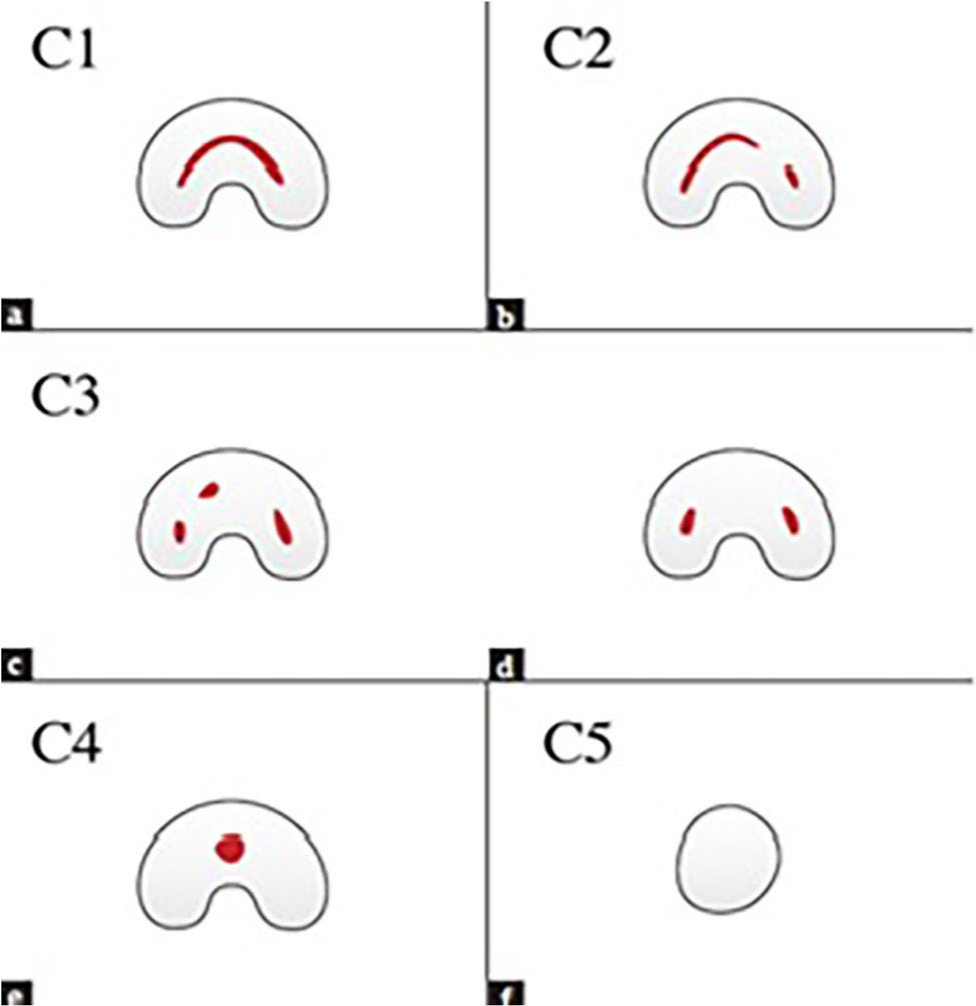
Classification of C-shaped canal configuration by Fan et al

The C-shaped canal morphology is based on the transverse section of the tooth root, with a shape like the letter C [2]. The primary cause of this anomaly is a defect in the attachment of Hertwig’s epithelial root sheath [1]. The presence of fins or isthmuses connecting root canals is the main anatomical feature of these roots. While its shape can be ribbon-shaped or have a curvature of 180° or more at the canal orifice, the 3-dimensional (3D) shape, and cross-section of the canal vary along the root length [3]. The canal shape at the orifice may differ from that in the middle or apical third [4]. C-shaped canals were first introduced by Cook and Cox in 1979 [1]. Fan et al. classified the cross-sectional shape of C-shaped canals into five categories (i.e., C1, C2, C3, C4, and C5), which are described below (Fig. 1) [4].

C1: Complete C without separation.

C2: Comma-shaped due to discontinued C.

C3: 2 or 3 separate canals.

C4: A round or oval canal in cross-section, usually near the apex.

C5: No visible canal lumen and usually observable near the apex.

The presence of a thin wall in C-shaped canals can cause problems in root canal treatment, including inadequate debridement, strip perforation, and missing canal [5]. In addition, it may ultimately affect the prognosis of treatment.

The anatomical diversity of the root has been investigated using various methods, such as tooth sectioning, microscopic evaluation, periapical radiography, micro-CT and cone beam computed tomography (CBCT) images [2]. CBCT is a useful and non-invasive method to examine root canal anatomy because 3D images allow for a more accurate assessment of root canal anatomy than conventional 2D radiographs, such as panoramic and periapical [5], depicting structures without distortion or superimposition [4].

Sufficient knowledge of the anatomical variations of the dental root canal and its prevalence in different teeth is essential for successful root canal treatment. Iatrogenic errors during operating such as missed canals, perforation, root canal transportation and improper root canal filling can be caused by insufficient knowledge of root canal morphology [2]. There are other factors involved and insufficient knowledge is one of the causes.

Several studies have evaluated the morphological features of C-shaped canals and the prevalence of their different types, reporting differences in their prevalence among different dental groups and populations [6]. Research on the prevalence and morphology of C-shaped canals in different teeth and populations has reported contradictory results. Therefore, this study seeks to more comprehensively investigate the prevalence and morphology of C-shaped canals as a systematic review.

A systematic review and meta-analysis by Martin et al. in 2019 provided valuable insights into the prevalence of c-shaped canals. After this study, many studies have investigated the prevalence of C-shaped canals in different populations, revealing the need for a new study to obtain comprehensive information about the prevalence of these canals. The study by Martin et al. did not provide a morphological classification of c-shaped canals. The main focus of the present study is to categorize c-shaped canals based on the classification by Fan et al. This study also focuses on analyzing the prevalence of C-shaped canals based on gender, geographical distribution, and anatomical location in the jaw, to gain a more accurate and comprehensive understanding of this anatomical variation.

## Materials and methods

This meta-analysis systematic review was approved by the Research Council of Hamadan University of Medical Sciences (ethical code: IR.UMSHA.REC.1403.517) and conducted using the Preferred Reporting Items for Systematic Reviews and Meta-Analysis (PRISMA) checklist [7]. The study protocol was registered in PROSPERO (with ID: CRD42024522710).

### Eligibility criteria

The population, intervention, comparison, and outcome (PICO) framework was applied to define eligibility criteria. The study population consisted of patients aged 15–95 years old with intact maxillary and mandibular posterior teeth, without caries or extensive restorations, or root canal-filling materials, having high-quality CBCT without artifacts. The intervention category was not applicable in this study. A comparison was made to demonstrate the prevalence of C-shaped canals in maxillary and mandibular posterior teeth. The outcome is the prevalence of c-shaped canals in premolar and molar teeth.

 Inclusion criteria:

1. Studies that examined the prevalence of C-shaped canals in maxillary and mandibular posterior teeth.
2. Studies that used CBCT to diagnose C-shaped canals.
3. Studies published in English up to 2024.

Exclusion criteria:

Review and meta-analysis studies.
In vitro studies.
3. Studies that utilized other imaging techniques, such as panoramic radiography, magnetic resonance imaging, and micro-computed tomography (micro-CT), to diagnose C-shaped canals.

## Data sources

PubMed, Web of Science, and Scopus databases were searched for studies published up to 2024.

## Search strategy

A separate search strategy was considered for each database using the main keywords (i.e., CBCT, C-shaped canal, premolar, and molar teeth) and their synonyms. PubMed, Scopus, and Web of Science databases were searched based on medical subject headings (MeSH), title/abstract keywords, and the subject, respectively (Table 1). A manual search was performed to find more articles in the reference lists of selected articles. A limited search was also conducted on the websites of related conferences held in the last 5 years. The search process was performed by two authors separately, and the selected articles were imported into EndNote 21 (Thomson Reuters, Toronto, and Canada). After eliminating duplicate articles in the next step, irrelevant articles were excluded by two authors by reading their titles and abstracts and focusing on the full text of the remaining articles. The required data were collected from the remaining articles after removing studies that were ineligible for the criteria. A third party was consulted in case of disagreement among the two authors, followed by finalizing the process of selecting the articles.

**Table 1:** The search strategy in databases

Pubmed: (“cbct“[all fields] OR “cone-beam computed tomography“[all fields] OR “cone beam computed tomography“[all fields] OR “cone beam computed tomography“[MeSH Terms]) AND (“tooth“[all fields] OR “tooth“[MeSH Terms] OR “root canal“[all fields] OR “root canal“[MeSH Terms] OR “molar“[all fields] OR “molar“[MeSH Terms] OR “premolar“[all fields] OR “premolar“[MeSH Terms]) AND (“anatomy“[all fields] OR “anatomy“[MeSH Terms] OR “morphology“[all fields] OR"morphology“[MeSH Terms] OR “c-shape“[all fields] OR “c-shaped“[all fields] OR “root canal shaped “[all fields] OR “root canal morphology “[all fields])
**WoS**: (“cbct“[all fields] OR “cone-beam computed tomography“[all fields] OR “cone beam computed tomography“[all fields] OR “cone beam computed tomography“[TOPIC]) AND (“tooth“[all fields] OR “tooth“[TOPIC] OR “root canal“[all fields] OR “root canal“[TOPIC] OR “molar“[all fields] OR “molar“[TOPIC] OR “premolar“[all fields] OR “premolar“[TOPIC]) AND (“anatomy“[all fields] OR “anatomy“[TOPIC] OR “morphology“[all fields] OR “morphology“[TOPIC] OR"c-shape“[all fields] OR “c-shaped“[all fields] OR “root canal shaped “[all fields] OR “root canal morphology “[all fields])
**Scopus**: (TITLE-ABS-KEY (“cbct”) OR TITLE-ABS-KEY (“cone-beam computed tomography”) OR TITLE-ABS-KEY(“cone beam computed tomography”)) AND (TITLE-ABS-KEY (“tooth”) OR TITLE-ABS-KEY (“root canal”) OR TITLE-ABS-KEY(“molar”) OR TITLE-ABS-KEY (“premolar”)) AND (TITLE-ABS-KEY (“anatomy”) OR TITLE-ABS-KEY (“morphology”) OR (TITLE-ABS-KEY (“c-shape”) OR TITLE-ABS-KEY (“c-shaped”) OR (TITLE-ABS-KEY (“root canal shaped “) OR TITLE-ABS-KEY(“root canal morphology “)

## Data items

The research question was answered by two authors extracting the information independently and completely (Table 1) which is shown in additional file 1.

## Risk of bias assessment

The articles were qualified using the Newcastle-Ottawa scale for cross sectional studies [8]. The correlation coefficient was used with a 95% confidence interval as an effect measurement. Sources of funding for the studies included in the review were not considered. The I^2^ statistic was utilized to determine heterogeneity, and comprehensive meta-analysis software was employed for the analysis. Subgroup analysis was based on tooth type, gender, left and right positions, geographical region, and C-shaped canal types according to Fan’s classification. Measurements are reported with a 95% confidence interval.

## Results

**Fig. 2:**
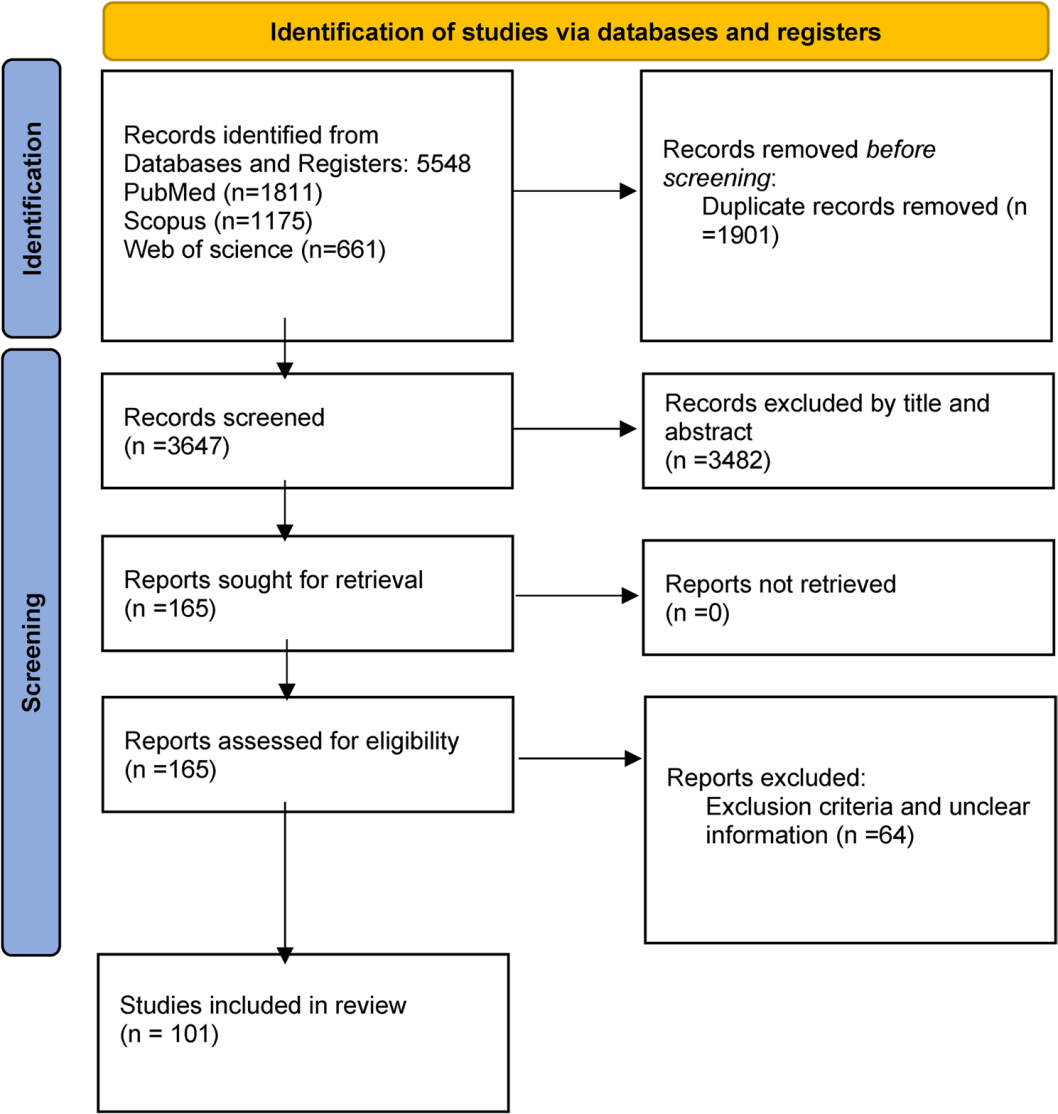
PRISMA flowchart for the systematic review

A total of 5,548 studies were retrieved from the initial search of three databases, of which 1,901 duplicate studies were excluded in the first step. Next, 3,649 studies were selected for reviewing titles and abstracts. After the review, 3,482 studies were excluded because their titles were irrelevant to the study topic and failed to meet the inclusion criteria in the abstracts. Finally, 165 studies were selected for full-text reviews; however, some of them were excluded based on the inclusion and exclusion criteria or because of insufficient data. After excluding 64 studies, 101 articles (published between 2011 and 2024) were finally selected for the meta-analysis (Fig. 2) [1, 2, 5, 6, 9–105].

Quality and bias risk of cross-sectional studies were measured using the Newcastle-Ottawa scale. The three domains in Newcastle-Ottawa adopted for cross-sectional studies are examined: selection (4 questions and maximum domain score = 5), comparison (one question and maximum domain score = 2) and outcome (2 questions and maximum score = 3) (Table 2). The quality assessment results from this checklist indicated that 101 studies were good (score 7–8). Heterogeneity analysis using the I^2^ statistic revealed that the heterogeneity of studies was 18.9% for maxillary first molar versus 84–98% for mandibular first premolar, mandibular second premolar, mandibular first molar, mandibular second molar, mandibular third molar, and maxillary second molar. Prevalence is dependent on location and time and varies with changes in each of these factors. Therefore, differences in results, even within the same community but at different times, are quite expected, and this causes heterogeneity in the data. It should be noted that, factors such as heredity, ethnic diversity and migration between different communities in modern times can also affect the prevalence and heterogeneity of the data. No studies were found regarding maxillary first premolar and second premolar. Only one study was related to maxillary third molar, which was excluded due to insufficient data.

**Table 2:** Risk of bias assessment in included studies using the Newcastle-Ottawa checklist for cross sectional studies

	author		Selection			Comparability		Outcome	
		Representativeness of the sample:	Sample size:	Non-respondents:	Ascertainment of the exposure (risk factor):	Comparability	Assessment of outcome:	Statistical test:	Total
1	E. Silva(2013) [10]	*			*	**	**	*	7
2	J. N. R. Martins(2017) [13]	*			*	**	**	*	7
3	M. von Zuben(2017) [16]	*			*	**	**	*	7
4	Y. C. Wu(2018) [19]	*			*	**	**	*	7
5	E. Kose and R. S. Ak(2021) [22]	*			*	**	**	*	7
6	M. Srinisha and K. Anjaneyulu(2020) [24]	*			*	**	**	*	7
7	H. Y. Ren(2020) [9]	*			*	**	**	*	7
8	X. Yu(2012) [29]	*			*	**	**	*	7
9	S. Mimica(2024) [32]	*			*	**	**	*	7
10	T. A. Fenelon (2022) [35]	*			*	**	**	*	7
11	D. B. S. Ladeira(2014) [38]	*			*	**	**	*	7
12	K. R. V. deAzevedo(2019) [41]	*	*		*	**	**	*	8
13	A. Shemesh(2017) [46]	*			*	**	**	*	7
14	Y. Y. Qian(2022) [49]	*			*	**	**	*	7
15	L. Yang(2022) [52]	*	*		*	**	**	*	8
16	S. M. Saber(2023) [6]	*			*	**	**	*	7
17	S. Khawaja(2021) [56]	*	*		*	**	**	*	8
18	A. Sinanoglu(2024)	*			*	**	**	*	7
19	J. N. R. Martins(2016) [12]	*			*	**	**	*	7
20	M. Shah(2023) [15]	*			*	**	**	*	7
21	Y. C. Chen(2018) [11]	*			*	**	**	*	7
22	E. M. Vega-Lizama(2021) [18]	*			*	**	**	*	7
23	D. Matus(2023) [21]	*				**	**	*	6
24	H. Yang(2013) [96]	*			*	**	**	*	7
25	F. Peña-Bengoa(2022) [26]	*	*		*	**	**	*	8
26	F. Peña-Bengoa(2021) [28]	*	*		*	**	**	*	8
27	M. Piskórz(2022) [31]	*			*	**	**	*	7
28	R. C. Piorno(2022) [24]	*			*	**	**	*	7
29	M. H. Mashyakhy(2020) [37]	*			*	**	**	*	7
30	F. Al-Sheeb(2022) [40]	*			*	**	**	*	7
31	N. Joshi(2021) [43]	*	*		*	**	**	*	8
32	E. M. Senan(2021) [45]	*			*	**	**	*	7
33	H. Ulfat(2021) [48]	*	*		*	**	**	*	8
34	S. Wadhwani(2017) [51]	*			*	**	**	*	7
35	Y. Nejaim(2020) [54]	*			*	**	**	*	7
36	G. Brea(2021) [55]	*			*	**	**	*	7
37	M. I. Almansour(2022) [58]	*			*	**	**	*	7
38	R. Zhang(2011) [97]	*			*	**	**	*	7
39	N. Riazifar(2018) [62]	*			*	**	**	*	7
40	R. P. Mohan(2017) [64]	*			*	**	**	*	7
41	Z. Donyavi(2019) [14]	*			*	**	**	*	7
42	G. D. Buchanan(2022) [17]	*	*		*	**	**	*	8
43	G. D. Buchanan(2023) [20]	*			*	**	**	*	7
44	J. Y. Y. Pan(2019) [23]	*			*	**	**	*	7
45	Y. E. Jang(2019) [25]	*	*		*	**	**	*	8
46	F. Gomez(2021) [27]	*			*	**	**	*	7
47	P. Thanaruengrong(2021) [30]	*	*		*	**	**	*	8
48	M. I. Karobari(2023), [33]	*	*		*	**	**	*	8
49	T. Al Omari(2022) [36]	*			*	**	**	*	8
50	C. Chen(2022) [39]	*	*		*	**	**	*	7
51	R. Shigefuji(2022) [42]	*			*	**	**	*	7
52	M. Shekarian(2023) [2]	*	*		*	**	**	*	8
53	Y. Alnowailaty(2022) [47]	*	*		*	**	**	*	8
54	H. Priyank(2023) [50]	*			*	**	**	*	7
55	J. B. Park(2013) [53]	*			*	**	**	*	7
56	E. Kantilieraki(2019) [44]	*			*	**	**	*	7
57	H. Aydın(2024) [57]	*	*		*	**	**	*	8
58	J. N. R. Martins(2016) [60]	*			*	**	**	*	7
59	H. Arslan(2015) [61]	*			*	**	**	*	7
60	Q. Zheng(2011) [98]	*			*	**	**	*	7
61	Z. S. Madani(2017) [65]	*			*	**	**	*	7
62	M. Janani(2018) [67]	*	*		*	**	**	*	8
63	S. Hiran(2021) [69]	*			*	**	**	*	7
64	L. M. M. Kenawi(2022) [71]	*			*	**	**	*	7
65	A. Torres(2015) [73]	*			*	**	**	*	7
66	T. Singh(2022) [75]	*			*	**	**	*	7
67	P. Somasundaram (2017) [76]	*			*	**	**	*	7
68	R. K. Yadav(2023) [77]	*			*	**	**	*	7
69	R. Arayasantiparb (2021) [78]	*			*	**	**	*	7
70	S. Demirbuga(2013) [79]	*			*	**	**	*	7
71	H. H. Jo(2016) [80]	*			*	**	**	*	7
72	H. S. Kim(2018) [81]	*			*	**	**	*	7
73	S. E. Yang(2021) [5]	*			*	**	**	*	7
74	K. Abdalrahman (2022) [1]	*			*	**	**	*	7
75	A. Nouroloyouni (2023) [82]	*	*		*	**	**	*	8
76	S. Sönmez Kaplan(2021) [83]	*			*	**	**	*	7
77	T. Funakoshi(2021) [84]	*			*	**	**	*	7
78	I. Kaya Büyükbayram (2019) [85]	*			*	**	**	*	7
79	M. Mashyakhy(2019) [86]	*			*	**	**	*	7
80	W. C. Ngeow (2020) [66]	*			*	**	**	*	7
81	H. Alfawaz(2019) [87]	*			*	**	**	*	7
82	B. Aricioğlu(2021) [88]	*			*	**	**	*	7
83	R. ChaintiouPiorno (2021) [89]	*			*	**	**	*	7
84	A. J. Alenezi(2022) [68]	*			*	**	**	*	7
85	D. S. Abdulateef (2021) [70]	*			*	**	**	*	7
86	S. Srivastava(2019) [90]	*	*		*	**	**	*	8
87	S. Y. Kim(2016) [91]	*			*	**	**	*	7
88	A. M. Pawar(2017) [92]	*			*	**	**	*	7
89	S. Živanovic(2021) [72]	*			*	**	**	*	7
90	E. Pedemonte(2018) [93]	*			*	**	**	*	7
91	D. G. Seo(2012) [94]	*			*	**	**	*	7
92	S. Suresh(2023) [63]	*	*		*	**	**	*	8
93	M. Feghali(2022) [99]	*			*	**	**	*	7
94	A. Haddadi(2019) [74]	*			*	**	**	*	7
95	D. Helvacioglu (2013) [100]	*			*	**	**	*	7
96	I. A. Sherwood (2019)	*			*	**	**	*	7
97	Y. C. Wu(2020) [101]	*			*	**	**	*	7
98	J. Abarca(2020) [102]	*			*	**	**	*	7
99	M. Tassoker(2018) [103]	*			*	**	**	*	7
100	Q. Guo(2023) [104]	*			*	**	**	*	7
101	O. Rae(2023) [105]	*	*		*	**	**	*	8

## Prevalence of C-shaped canals in different teeth

**Fig. 3:**
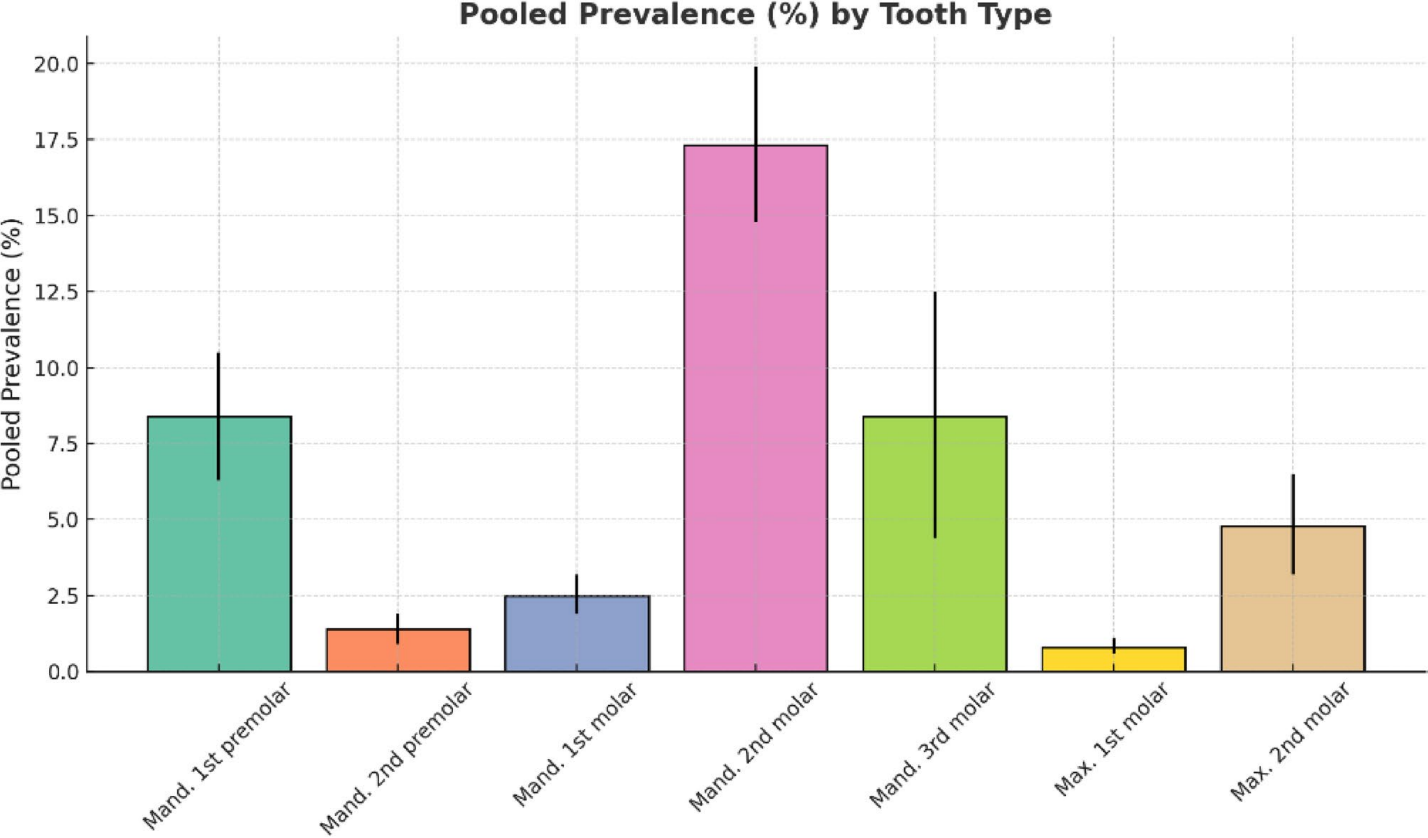
Pooled prevalence (%) of C-shaped canal configuration across different tooth type

**Fig. 4:**
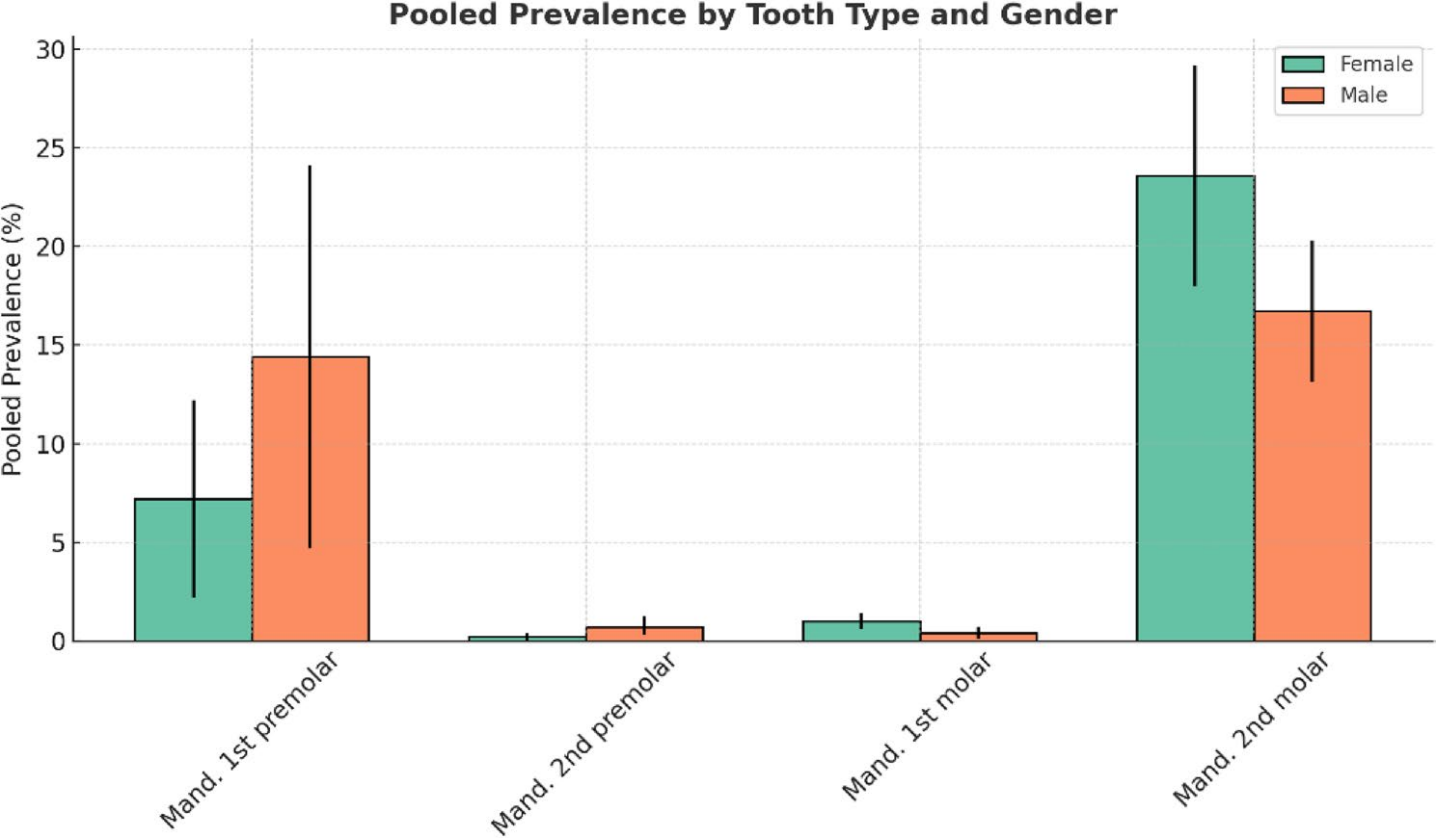
Grouped bar chart showing the pooled prevalence (%) of C-shaped canal by tooth type and gender

**Fig. 5:**
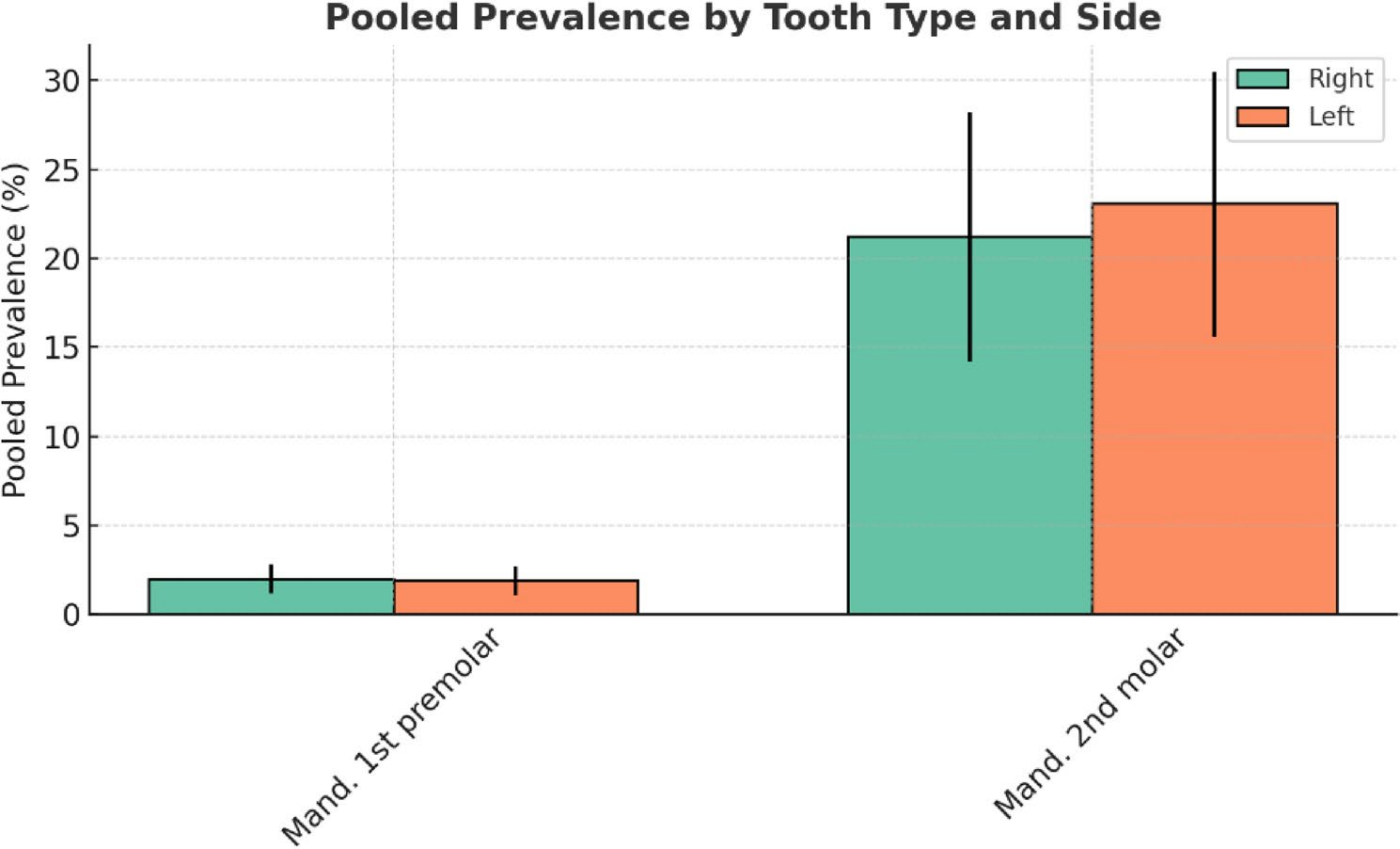
Grouped bar chart comparing pooled prevalence (%) of C-shaped canal between right and left for each tooth type

Based on the meta-analysis results according to the tooth type (Table 3; Fig. 3), the highest and lowest prevalence rates of the C-shaped canal were 95% CI: 14.8–19.9% and 95% CI: 0.6–1.1% in mandibular second molar and maxillary first molar, respectively. The forest plot diagram for each tooth is presented in Figs. 1, 2, 3, 4, 5, 6 and 7 which is shown in appendix file.

**Table 3:** C-shaped Canal prevalence in different teeth

	Tooth type	Pooled prevalence	Odds ratio 95% Conf. interval	Number of studies
**1**	Mandibular first premolar	8.4%	6.3–10.5%	19
**2**	Mandibular second premolar	1.4%	0.9–1.9%	16
**3**	Mandibular first molar	2.5%	1.9–3.2%	19
**4**	Mandibular second molar	17.3%	14.8–19.9%	75
**5**	Mandibular third molar	8.4%	4.4–12.5%	5
**6**	Maxillary first molar	0.8%	0.6–1.1%	7
**7**	Maxillary second molar	4.8%	3.2**–**6.5%	12

## Prevalence of the C-shaped Canal by gender

Based on the results of the statistical analysis (Table 4; Fig. 4), the C-shaped canal in mandibular first and second premolars was more prevalent in men than in women. In addition, its prevalence in mandibular first and second molars was higher in women than in men. Due to insufficient studies, the mandibular third molar, maxillary first molar, and maxillary second molar were not included in the gender-based statistical analysis. Figures 8, 9, 10 and 11 illustrate the forest plot diagram for each tooth which is shown in appendix file.

**Table 4:** The C-shaped Canal prevalence by gender in different teeth

	Tooth type	Gender	Pooled prevalence	Odds ratio 95% Conf. interval
1	Mandibular first premolar	F	7.2%	2.2–12.2%
		M	14.4%	4.7–24.1%
2	Mandibular second premolar	F	0.2%	0–0.4.4%
		M	0.7%	0.3–1.2%
3	Mandibular first molar	F	1%	0.6–1.4%
		M	0.4%	0.1–0.7%
4	Mandibular second molar	F	23.6%	18–29.2.2%
		M	16.7%	13.1–20.3%

### Prevalence of the C-shaped Canal by right and left sides

Given the availability of sufficient studies, the mandibular first premolar and mandibular second molar were included in the statistical analysis (Table 5; Fig. 5). The prevalence of C-shaped canals did not differ significantly in the mandibular first premolar and mandibular second molar on the right and left sides. The forest plot diagram for each tooth is provided in Figs. 12 and 13 which is shown in appendix file.

**Table 5:** The prevalence of C-shaped canals on the right and left sides

	Tooth type	Right/left	Pooled prevalence	Odds ratio 95% Conf. interval
1	Mandibular first premolar	Right	2%	1.2–2.8%
		Left	1.9%	1.1–2.7%
2	Mandibular second molar	Right	21.2%	14.2–28.2%
		Left	23.1%	15.6–30.5%

### Evaluation of C-shaped canals based on Fan’s classification in coronal, middle, and apical cross-sections of the root canal

The C-shaped canal classification was statistically analyzed for the mandibular first premolar, first molar, and second molar (Table 6). The highest prevalence of C-shaped canals in the mandibular first premolar was C3 in coronal, middle, and apical sections. In the coronal section of the mandibular second molar, C2 displayed the highest prevalence of 95% CI: 25.3–27.8%. In the middle and apical sections, C3 was most prevalent. Figures 14, 15, 16, 17, 18, 19, 20, 21 and 22 depict the forest plot diagram for each tooth which is shown in appendix file.

**Table 6:** Prevalence of C-shaped canals according to fan’s classification

	Tooth type	Cross section	Pooled prevalence	Odds ratio 95% Conf. interval
1	Mandibular first premolar	Coronal C1	0	0
		Coronal C2	1.9%	0–4.8.8%
		Coronal C3	14.8%	10.6–19%
		Coronal C4	8.3%	7.85–8.75%
		Middle C1	16.3%	11.8–20.8%
		Middle C2	30.5%	25.6–35.4%
		Middle C3	46.6%	41–52%
		Middle C4	6.1%	3.4–8.8%
		Apical C1	4.9%	2.5–7.2%
		Apical C2	20.6%	16.9–24.3%
		Apical C3	31.2%	26–36.3.3%
		Apical C4	9.4%	6.1–12.7%
		Apical C5	0.5%	0–1.2.2%
2	Mandibular first molar	Coronal C1	67.5%	60.4–74.5%
		Coronal C2	3.2%	0.6–5.8%
		Coronal C3	2.2%	0.1–4.4%
		Coronal C4	24%	17.4–30.6%
		Middle C1	%25.1	31.5−18.8%
		Middle C2	%65	72.1−57.9%
		Middle C3	%3.4	0.8–6.1%
		Middle C4	0	0
		Apical C1	%39.1	32.2–46%
		Apical C2	4.2%	1.3-7.1%
		Apical C3	%42.1	34.9-49.3%
		Apical C4	%50.4	24.6-76.2%
3	Mandibular second molar	Coronal C1	%1.8	2.1-5.5%
		Coronal C2	%26.6	25.3-27.8%
		Coronal C3	%11.6	10.7-12.5%
		Coronal C4	%13.2	12-14.4%
		Middle C1	16%	16.9-17.1%
		Middle C2	25.1%	23.9-26.4%
		Middle C3	39.4%	38-40.8%
		Middle C4	1.3%	1-1.7%
		Middle C5	0.1%	0-0.3%
		Apical C1	12.4%	11.4–13.4%
		Apical C2	7.9%	7.1-8.7%
		Apical C3	39.9%	38.5-41.2%
		Apical C4	%17	15.9-18.1%
		Apical C5	%1.9	1.2-2.6%

### Prevalence of C-shaped canals by continents

Table 7 presents the articles based on the sufficient number of studies conducted in different continents for meta-analysis. Studies on mandibular second molars in different continents revealed that C-shaped canals were mostly prevalent in Asia 95% CI: 15.2–22.1%. The forest plot diagram for each tooth is illustrated in Figs. 23, 24, 25, 26 and 27 which is shown in appendix file.

**Table 7:** Prevalence of C-shaped canals according to different continents

	Tooth type	Continent	Pooled prevalence	Odds ratio 95% Conf. interval
1	Mandibular first premolar	America	16.4%	2.7–30%
		Asia	6.6%	2.8–10.4%
2	Mandibular second premolar	America	7.1%	4.3–10%
		Asia	1.2%	0.7–1.7%
3	Mandibular first molar	America	5.3%	2– 8.5 %
		Asia	2.1%	1.4–2.8%
4	Mandibular second molar	America	15.1%	11–19.3%
		Asia	18.6%	15.2–22.1%
		Africa	17.3%	14.7–19.8%
		Europe	10.7%	8–13.4%
	Maxillary first molar	Asia	0.7%	0.4–0.9%
5		Europe	1.1%	0.6–1.5%

### Sensitivity analysis

A leave-one-out sensitivity analysis was conducted to assess the robustness of the pooled prevalence estimates for each tooth group (Figs. 28, 29, 30, 31, 32, 33, 34, 35, 36 and 37 ) which is shown in appendix file.

For mandibular fires molars, exclusion of Shemesh et al. (2017) slightly changed the pooled prevalence estimate, which remained within the confidence intervals of the other studies. Given its large sample size (1299), excluding it would result in loss of valuable data.

In the mandibular first premolar subgroup, exclusion of R. Arayasantiparb (2021) increased the pooled prevalence estimate to 0.0434, suggesting it may have contributed to heterogeneity.

For mandibular third molars, the highest change was observed when M.Shekarian (2023) was excluded, increasing the estimate to 0.068. The relatively wide confidence intervals suggest an uncertainty in the pooled prevalence.

For maxillary second molar, excluding Z. Donyavi (2019) increased the pooled prevalence to 0.036, indicating this study notably impact on the results.

## Discussion

Being informed about the morphological diversity of root canals is an important factor in increasing the success rate of root canal treatment for dentists. Knowing these issues reduces treatment-related incidents, such as inadequate debridement, perforation, missed canals, and improper root canal filling. The morphological diversity of root canals is examined using various methods, such as conventional radiographs, micro-CT, and CBCT. CBCT is used to identify abnormal morphology and complex variations of root canals, considering its high accuracy and recommendation by the American Association of Endodontic and the American Academy of Oral and Maxillofacial Radiology [55].

The heterogeneity was generally high in articles reviewed in our study, as indicated by the I² statistics. This issue was due to potential sources of heterogeneity (e.g., differences in study populations, imaging protocols, diagnostic criteria, operator experience). Furthermore, the observed heterogeneity across studies may be attributed to time and location based variations. Even within the same population, differences over time may occur due to environmental changes or methodological inconsistencies. In addition, Genetic, ethnic diversity, and migration patterns may influence the reported prevalence. Therefore, a certain degree of heterogeneity is expected in systematic reviews involving diverse populations.

The results of our meta-analysis indicated the highest prevalence of C-shaped canals in the mandibular second molars, probably due to the high prevalence of anatomical abnormalities and root fusion in this tooth [1, 23], which is consistent with the results of the systematic review conducted by Martin et al. [3]. After the mandibular second molar, the C-shaped canal is most prevalent in the mandibular first premolar and mandibular third molar. In the maxilla, the prevalence of C-shaped canals is higher in the maxillary second molar than in the maxillary first molar.

The results of four studies confirmed that C-shaped canals in the first premolar were more prevalent in men [19, 39, 70, 88]. Based on the results of three studies, the prevalence of C-shaped canals in the second premolar was higher in men [39, 70, 88]. In five studies, the prevalence of C-shaped canals in the mandibular first molar was higher in women [2, 39, 47, 79, 88]. The results of 33 studies revealed that C-shaped canals in the mandibular second molar were more prevalent in women, which conforms to the findings of the systematic review performed by Martin et al. [1–3, 5, 6, 9, 14, 16, 18, 23, 27, 38, 39, 42–48, 51–53, 57, 58, 63, 77, 79, 81, 87, 88, 91, 92, 98]. Given the abundance of studies conducted on the mandibular second molar, the presence of C-shaped canals in the mandibular second molar is more prevalent in women. Therefore, more studies should be performed on other teeth. Gender-related differences in canal morphology may also reflect underlying biological or developmental factors, such as differences in craniofacial anatomy, hormonal influences during tooth development, or skeletal characteristics between sexes. However, further genetic and developmental studies are needed to better clarify these association.

The results of three studies demonstrated that the prevalence of C-shaped canals in the mandibular first premolar was not statistically significant on the right and left sides [19, 70, 96]. A similar prevalence of C-shaped canals in the mandibular second molar on the right and left sides was reported in 15 studies [1, 5, 9, 16, 23, 24, 45, 46, 53, 57, 58, 66, 68, 91, 98]. Overall, clinicians can consider the possibility of a C-shaped canal on the opposite side if they encounter the presence of a C-shaped canal during root canal therapy on one side.

The results of three studies showed that the prevalence of C3 type was higher in the mandibular first premolar in all three coronal, middle, and apical sections based on Fan’s classification [25, 39, 105]. In the mandibular second molar, the C2 type was observed more frequently in the coronal section and the C3 type in the middle and apical sections, as claimed in 31 studies [6, 9, 15, 18, 20, 27, 32, 35, 36, 39, 43–48, 59, 60, 67, 68, 72, 82, 83, 86, 87, 91, 92, 95, 98–100].

According to the results, the highest and the lowest prevalence rates of C-shaped canals in the mandibular second molar belonged to Asia and Europe, respectively. These disparities may be partially attributed to ethnic and genetic factors. It has been hypothesized that long-term evolutionary and environmental influences such as dietary habits and patterns of human migration may have contributed to morphological differences in root canal anatomy. However, these associations require further scientific validation.

In this respect, one study suggested a link between human gatherings in prehistoric times and morphological differences among current geographical regions [54].

Preoperative knowledge of root canal morphology is effective in root canal management. Dentists should maintain a high index of suspicion for C-shaped canal configurations, particularly in mandibular second molars and specific populations (e.g., females, Asian patients) If the presence of a C-shaped canal is suspected in conventional radiographs (e.g., panoramic and periapical), CBCT can be used for the 3D assessment of the root canal. According to the 2019 position statement of the European Society of Endodontology, CBCT is indicated in cases where conventional radiographs are insufficient, particularly for the assessment of complex root canal anatomy, identification of additional or missed canals, and pre-surgical planning in endodontics [106]. As previously stated, CBCT has several advantages: It is non-invasive method, the morphology of root canal can be examined in orthogonal planes(coronal, axial and sagittal) due to the lack of superimposition of anatomical structures, improves the field of view, provides high-resolution images, and requires less radiation compared to conventional computed tomography. To reduce radiation dose, it is recommended to use a smaller field of view and reducing the exposure parameters as much as possible [23]. Access cavity preparation is considerably different in the tooth with C-shaped canals. More effective methods, such as ultrasonic activation, can be utilized for deeper penetration of the canal irrigation material to clean the root canal. The most commonly root canal obturation techniques include cold lateral compaction, core carrier and warm vertical compaction. In the cold lateral compaction technique, root canal obturation is achieved by laterally compaction of the accessory cones against a master cone. In the core carrier technique, a heat-softened gutta-percha coated on a carrier is used. As in the continuous wave obturation technique, thermoplasticizied guttapercha is injected into the root canal and compacted [107]. The root canal obturation technique may require some modification. The presence of an isthmus in the C-shaped canal may prevent sufficient flaring or deep placement of the spreader in the lateral condensing method [108, 109]. It has been shown that the core carrier and continuous wave techniques can be successful in filling abnormal root canals due to their conformability to the canal walls. The results of the recent study emphasize the importance of choosing the obturation technique in different types of c-shaped canals based on the classification of Fan et al., as it states that in C2, the continuous wave technique and in C3, the core carrier technique has a smaller gap in root canal filling [107].

Sensitivity analysis indicated that the overall estimates were stable. While exclusion of certain studies (e.g., R. Arayasantiparb 2021, Z. Donyavi 2019, C. Chen 2022 and Shemesh et al. 2017) altered the pooled prevalence.

The limitations of this study include the potential impact of publication bias, exclusion of non-English studies, and reliance on cross-sectional studies, which may have limited the ability to infer causality. The exclusion of certain studies may have led to underestimation of the prevalence of C-shaped canal in various populations and could have significantly influenced the results of the meta-analysis. Future studies should implement broader search strategies, including grey literature and unpublished data, to reduce the risk of selection bias. Additionally larger sample sizes and more robust study designs are needed to improve the validity and generalizability of the findings. Variability in CBCT resolution across the included studies may have led to diagnostic misclassification of C-shaped canals. Moreover, inconsistency in the application and interpretation of Fan’s classification may have contributed to heterogeneity in the reported canal types.

Given the prevalence of C-shaped canals, especially in mandibular second molars, it is suggested that future studies compare the impact of different imaging techniques (e.g., CBCT vs. 2D conventional radiographs) on the detection and management of C-shaped canals. Moreover, it is recommended that longitudinal studies explore the long-term outcomes of root canal treatment in teeth with C-shaped canals.

## Conclusion

The findings of this systematic review and meta-analysis confirmed the significant prevalence of C-shaped canals in mandibular second molars compared to other teeth. The highest prevalence of C-shaped canals in mandibular second molars was observed in Asia. After the mandibular second molars, the highest prevalence of C-shaped canals belonged to the mandibular first premolars and mandibular third molars. Overall, it is necessary to obtain sufficient information about the prevalence and complex morphology of these canals for successful root canal therapy. Furthermore, using advanced imaging techniques (e.g., CBCT) plays a decisive role in detecting these canals.

## Supplementary Information

Supplementary Material 1.

Supplementary Material 2.

## Data Availability

The datasets used and/or analysed during the current study are available from the corresponding author on reasonable requestpoint of contact: Dr. Elham Shokri, email: elahmshokri@gmail.com.

## Abbreviations

CBCT: Cone-beam computed tomography
PRISMA: Preferred Reporting Items for Systematic Reviews and Meta-Analysis
PICO: Population, Intervention, Comparison, Outcome
micro-CT: micro-Computed tomography
MeSH: medical subject headings
WoS: Web of Science
CI: Confidence interval
