# Supplementary material for: Prevalence of c-shaped canal morphology in premolar and molar teeth assessed by cone-beam computed tomography: systematic review and meta-analysis
Source: BMC Oral Health. 2025 Oct 22;25:1657. doi: 10.1186/s12903-025-06946-8 (PMC12542057; doi:10.1186/s12903-025-06946-8)
Supplement: Supplementary file 1 — Supplementary Material 1. [file 12903_2025_6946_MOESM1_ESM.docx]

|  | |  |  | | | | | | |  |  |  |  |  |  |  |  |  |  |  |  |  |  |  |
| --- | --- | --- | --- | --- | --- | --- | --- | --- | --- | --- | --- | --- | --- | --- | --- | --- | --- | --- | --- | --- | --- | --- | --- | --- |
|  | **Author** | year | Country | patient | | | | | | CBCT Device | | Voxel size mm | Age average | LPM1 | LPM2 | LM1 | LM2 | LM3 | UM1 | UM2 | Number of c-shaped/ number of teeth(female)  Prevalace% | Number of c-shaped/ number of teeth(male)  Prevalace% | Number of c-shaped/ number of teeth(right)  Prevalace% | Number of c-shaped/ number of teeth(left) |
|  |  |  |  | F | | | | | M |  |  |  |  |  |  |  |  |  |  |  |  |  |  | Prevalance% |
| 1 | E. Silva | 2013 | Brazil | 84 | | | | | 70 | iCAT | | 0.2 |  |  |  | 234/4  1.7% | 226/8  3.5% |  |  |  |  |  |  |  |
| 2 | J. N. R. Martins | 2017 | Portugal | 402 | | | | | 232 | planmeca | | 0.2 | 50 | 1123/26  2.3% | 889/5  0.6% |  |  |  |  |  | nc | nc | nc | nc |
| 3 | M. von Zuben | 2017 | Portugal | 2735 | | | | |  | Variable | | 0.25 | 43 |  |  |  | 3600/499 |  |  |  | 2039/336  16.5% | 1561/162  10.4% | 1712/245  14.3% | 1888/244  12.9% |
| 4 | Y. C. Wu | 2018 | Taiwan | 164 | | | | | 236 | newtom | | 0.15 | 40.4±15.6 | 800/92  11.5% |  |  |  |  |  |  | 328/29  8.8% | 472/63  13.3% | 400/47  11.7% | 400/45  11.2% |
| 5 | E. Kose and R. S. Ak | 2021 | Turkey | 286 | | | | | 231 | planmeca | | 0.2 | 34.2 |  |  |  |  |  | 709/7  1% | 739/36  4.8% | nc | nc | nc | nc |
| 6 | M. Srinisha and K. Anjaneyulu | 2020 | *India* | 50 | | | | |  |  | |  |  |  |  |  | 100/3  3% |  |  |  |  |  | 50/1  1% | 50/2  2% |
| 7 | H. Y. Ren | 2020 | China | 39 | | | | |  | Newtom | | 0.3 | 28.82 |  |  |  | 68/28  41.18% |  |  |  | 31/12  38.71% | 37/16  43.24% | 34/13  38.24% | 34/15  44.12% |
| 8 | X. Yu | 2012 | China | 149 | | | | |  | morita | | 0.125 |  | 178/2  1.1% | 178/1  0.6% |  |  |  |  |  |  |  | nc | nc |
| 9 | S. Mimica | 2024 | Switzerland | 608 | | | | |  | soredex | | 0.2 | 30 |  |  |  | 859/78  9.1% |  |  |  |  |  |  |  |
| 10 | T. A. Fenelon | 2022 | Australia | 458 | | | | | 199 | morita | | 0.125 | 23 |  |  |  | 1278/163 |  |  |  | nc | nc |  |  |
| 11 | D. B. S. Ladeira | 2014 | Brazil | 130 | | | | | 84 | iCAT | | 0.25 | 29.9 |  |  |  | 406/62  15.3% |  |  |  | 248/43 | 158/19 |  |  |
| 12 | K. R. V. de Azevedo | 2019 | Brazil | 192 | | | | | 142 | iCAT | | 0.2 | 19_95 |  |  | 379/91  24.01% | 422/90  21.32% |  |  |  |  |  |  |  |
| 13 | A. Shemesh | 2017 | Israel | 573 | | | | | 447 | Alioth | | 0.155 | 43.05 |  |  | 1229/2  0.16% | 1465/68  4.6% |  |  |  | LM2  818/42  5.1% | LM2  647/26  4% | LM2  739/34  4.6% | LM2  726/34  4.7% |
| 14 | Y. Y. Qian | 2022 | China | 1508 | | | | |  | Morita | | 0.125  0.250 | 38.56±14.38 |  |  |  |  |  | 1488/8  0.54% | 1547/81  5.24& | nc | nc | nc | nc |
| 15 | L. Yang | 2022 | China | 896 | | | | |  | Newtom | | 0.25 |  |  |  |  | 1200/430  35.8% |  |  |  | 600/255  42.5% | 600/175  29.1% |  |  |
| 16 | S. M. Saber | 2023 | Egypt |  | | | | |  | Planmeca | | 0.075 | 15_65 |  |  |  | 350/45  12.9% |  |  |  | 215/29 | 135/16 |  |  |
| 17 | S. Khawaja | 2021 | United Arab Emirates | 139 | | | | | 115 | Sirona | | 0.15 | 15_75 |  |  |  | 508/91  17.9% |  |  |  |  |  |  |  |
| 18 | A. Sinanoglu | 2014 | Turkey | 106 | | | | | 94 | iCAT | | 0.25 | 35 |  |  |  | 339/29  8.6% |  |  |  |  |  |  |  |
| 19 | J. N. R. Martins | 2016 | Portugal | 585 | | | | | 310 | Planmeca | | 0.2 | 52 |  |  |  |  |  | 928/10  1.1% | 1299/49  3.8% | nc | nc | nc | nc |
| 20 | M. Shah | 2023 | Pakistan |  | | | | | | Care stream | | 1 |  |  |  |  | 150/13  8.7% |  |  |  | nc | nc |  |  |
| 21 | Y. C. Chen | 2018 | Taiwan | 317 | | | | | | Newtom | | 0.15 |  |  | 580/13  2.24% |  |  |  |  |  |  |  |  |  |
| 22 | E. M. Vega-Lizama | 2021 | Mexico | 335 | | | | | 190 | iCAT | | 0.2 | 37.73 |  |  |  | 1050/225 |  |  |  | 670/158 | 380/67 |  |  |
| 23 | D. Matus | 2023 | Chile |  | | | | | | vatech | | 0.12 |  |  |  | 351/8  2.3% |  |  |  |  |  |  |  |  |
| 24 | H. Yang | 2013 | China | 238 | | | | | | Galileos | | 0.125 |  | 440/5  1.14% |  |  |  |  |  |  |  |  | 223/2 | 217/3 |
| 25 | F. Peña-Bengoa | 2022 | Chile | 226 | | | | | | iCAT | |  |  |  |  | 456/3 | 456/67 |  |  |  | nc | nc |  |  |
| 26 | F. Peña-Bengoa, | 2021 | Chile | 226 | | | | | | iCAT | |  |  |  |  | 456/0 | 456/69 |  |  |  | nc | nc |  |  |
| 27 | M. Piskórz | 2022 | *Poland* | 19 | | | | | | Dürr Dental | |  | 27.6 |  |  |  | 37/21  56.8% |  |  |  | nc | nc |  |  |
| 28 | R. C. Piorno | 2022 | *Argentina* | 69 | | | 51 | | | Planmeca | | 0.15  0.2 | 40 |  |  |  |  |  | 120/5  4% | nc | nc | na | nc | nc |
| 29 | M. H. Mashyakhy | 2020 | Saudi Arabia | 108 | | | 100 | | | morita | | 0.25 | 26 | 397/6  1.5% | 379/3  0.8% | 290/0 | 367/29  7.9% |  |  |  |  |  | PM  385/6 | PM  391/3 |
|  |  |  |  |  |  |  |  |  |  |  |  |  |  |  |  |  |  |  |  |  |  |  | M  331/12 | M  326/17 |
| 30 | F. Al-Sheeb | 2022 | Qatar |  | | | | | | iCAT | | 0.2  0.4 | 34.4 |  |  |  | 255/7  2.8% |  |  |  |  |  |  |  |
| 31 | N. Joshi | 2021 | Nepal |  | | | | | | Planmeca | |  |  |  |  |  | 199/25  12.6% |  |  |  | 91/14  15.4% | 108/11  10.2% | 98/11  12.22% | 101/14  13.9% |
| 32 | E. M. Senan | 2021 | Yemen | 125 | | | 125 | | | Vatech | | 0.12 | 18_40 |  |  |  | 500/45  9% |  |  |  | 250/27 | 250/18 | 250/22 | 250/23 |
| 33 | H. Ulfat | 2021 | Pakistan | 183 | | | 177 | | | Planmeca | | 0.2 | 17_70 |  |  |  | 720/72  10% |  |  |  | 366/x  15.6% | 354/x  4.2% |  |  |
| 34 | S. Wadhwani | 2017 | India | 238 | | | | | | KODAK | |  |  |  |  |  | 238/23  9.7% | 238/19  8% |  |  | LM2  142/16  LM3  142/15 | LM2  96/7  LM3  96/4 | nc | nc |
| 35 | Y. Nejaim | 2020 | Brazil | 220 | | | 184 | | | iCAT | | 0.2 | 15_80 |  |  | 710/17  2.39% | 754/108  14.32% |  |  |  | nc | nc |  |  |
| 36 | G. Brea | 2021 | Venezuela | 292 | | |  | | | Care stream | | 0.2 |  | 380/110  28.94% | 308/22  7.14% |  |  |  |  |  | nc | nc |  |  |
| 37 | M. I. Almansour | 2022 | Saudi Arabia |  | | | | | | Care stream | | 0.075 | 18_ 65 |  |  |  | 304/13  4.3% |  |  |  |  |  |  |  |
| 38 | R. Zhang | 2011 | China | 110 | | | | | 101 | Morita | | 0.125 | 37 |  |  | 232/0 | 157/46  29% |  |  |  |  |  |  |  |
| 39 | N. Riazifar | 2018 | Iran |  | | | | | | Planmeca | | 0.15 |  |  |  |  | 550/75  13.6% |  |  |  |  |  |  |  |
| 40 | R. P. Mohan | 2017 | India |  | | | | | | Planmeca | |  |  |  |  |  |  |  | 143/0 | 139/2  1.4% |  |  |  |  |
| 41 | Z. Donyavi | 2019 | Iran | 301 | | | | | 201 | sordex | | 0.2 | 39.06±12.72 |  |  |  | 447/41  9.2% |  |  | 635/1 | LM2  275/29  10.5% | LM2  172/12  7% |  |  |
| 42 | G. D. Buchanan | 2022 | South Africa | 232 | | | | | | planmeca | | 0.15  _0.6 | 36.4 | 386/43  11.1% | 386/19  4.9% |  |  |  |  |  |  |  |  |  |
| 43 | G. D. Buchanan | 2023 | South Africa | 239 | | | | | | planmeca | | 0.15  _0.6 | 35.4 |  |  |  | 386/22  5.7% |  |  |  |  |  |  |  |
| 44 | J. Y. Y. Pan | 2019 | Malaysia | 118 | | | | | 90 | KOVO | | 0.25 | 28.7 |  |  |  | 376/173  48.7% |  |  |  | 216/117  54.2% | 160/66  41.3% | 185/89  48.1% | 191/94  49.2% |
| 45 | Y. E. Jang | 2019 | South Korea | 252 | | | | | 248 | Willmed | | 0.167 | 28.61±10.02 | 971/36  3.7% | 997/0 |  |  |  |  |  |  |  |  |  |
| 46 | F. Gomez | 2021 | Venezuela | 161 | | | | | | Care stream | |  |  |  |  |  | 190/37  19.5% |  |  |  | 109/18 | 81/19 |  |  |
| 47 | P. Thanaruengrong | 2021 | Thailand | 201 | | | | | 148 | morita | | 0.25 |  | 621/147  23.7% | 538/4  0.7% |  |  |  |  |  |  |  |  |  |
| 48 | M. I. Karobari, | 2023 | Saudi Arabia | 500 | | | | | | iCAT | | 0.2 |  | 645/0 | 585/5  0.4% |  |  |  |  |  |  |  | LPM2  293/2 | LPM2  292/3 |
| 49 | T. Al Omari | 2022 | Jordan | 2037 | | | | |  | Care stream | | 0.2 | 40 |  |  |  | 2845/342  12% |  |  |  | nc | nc | nc | nc |
| 50 | C. Chen | 2022 | China | 692 | | | | | 308 | Newtom | | 0.3 |  | 2000/205  10.25% | 2000/5  0.25% | 2000/11  0.55% | 2000/941  47.05% |  |  |  | Pm1  1384/105  Pm2  1384/2  M1  1384/9  M2  1384/732 | Pm1  616/100  Pm2  616/3  M1  616/2  M2  616/209 |  |  |
| 51 | R. Shigefuji | 2022 | Japan | 84 | | | | | 89 | Morita | |  | 40.4 |  |  |  | 173/61  35.3% |  |  |  | 84/39  46.6% | 89/22  24.7% |  |  |
| 52 | M. Shekarian, | 2023 | Iran | 151 | | | | | 141 | Sirona | | 0.18 |  |  |  | 291/5  1.7% | 402/8  2% | 200/4  2% |  |  | LM1  145/5  LM2  205/6  LM3  100/2 | LM1  146/0  LM2  205/2  LM3  54/2 |  |  |
| 53 | Y. Alnowailaty | 2022 | Saudi Arabia | 150 | | | | | 150 | iCAT | | 0.125 | 38.7±17.9 |  |  | 600/146  24.33% | 600/182  30.33% |  |  |  | LM1  300/78  LM2  300/75 | LM1  300/68  LM2  300/107 |  |  |
| 54 | H. Priyank | 2023 | India | 264 | | | | | | carestream | | 0.18 | 38.64±5.71 |  |  |  |  | 277/21  7.5% |  |  |  |  |  |  |
| 55 | J. B. Park | 2013 | Korea | 236 | | | 194 | | | M-view | | 0.4 | 38.1±18 | 797/0 | 789/0 | 726/0 | 710/293  41.3% |  |  |  | 373/151 | 337/142 | 361/154 | 349/139 |
| 56 | E. Kantilieraki | 2019 | Greece |  | | | | | | Newtom  sordex | | 0.1 | 37 |  |  | 478/0 | 524/53 |  |  |  | 317/36 | 207/17 |  |  |
| 57 | H. Aydın | 2024 | *Türkiye* | 215 | | | | | 215 | Sirona | | 0.16 |  |  |  |  | 860/83  9.65% |  |  |  | 430/45 | 430/38 | 430/44 | 430/39 |
| 58 | J. N. R. Martins | 2016 | Portugal | 489 | | | | | 303 | planmeca | | 0.2 | 51 |  |  | 695/4  0.6% | 1088/93  8.5% |  |  |  | LM2  690/77 | LM2  398/16 | LM2  538/51 | LM2  550/42 |
| 59 | H. Arslan | 2015 | *Türkiye* | 47 | | | | | 41 | Newtom | | 0.15 | 35.5 | 154/4  2.5% | 133/2  1.5% |  |  |  |  |  |  |  |  |  |
| 60 | Q. Zheng | 2011 | China | 608 | | | | |  | Morita | | 0.125 | 40.1 |  |  |  | 528/204  38.6% |  |  |  | 231/96 | 297/108 | 216/84 | 312/120 |
| 61 | Z. S. Madani | 2017 | Iran | 110 | | | | | | Newtom | | 0.3 |  |  |  | 154/2  1.2% | 147/26  17.6% |  |  |  | nc | nc |  |  |
| 62 | M. Janani | 2018 | Iran | 137 | | 94 | | | | Newtom | |  | 15_65 |  |  |  | 384/82  21.4% |  |  |  |  |  |  |  |
| 63 | S. Hiran | 2021 | *Thailand* | 137 | | 111 | | | | Morita | | 0.08  0.25 | 45 |  |  | 256/1  0.39% | 311/132  42.4% | 186/31  16.7% |  |  | nc | nc |  |  |
| 64 | L. M. M. Kenawi | 2022 | Saudi Arabia | 208 | | | | | | iCAT | | 0.25 | 18_ 55 |  |  |  | 283/X  5.7% | 347/X  4% |  |  |  |  |  |  |
| 65 | A. Torres | 2015 | *Belgium* | 100 Belgium | | | | | | Morita | | 0.25 | Belgium  19.5 |  |  | 0 | 112/12  10.71% |  |  |  |  |  |  |  |
|  |  |  |  | 170  Chile | | | | | |  |  |  | Chile  19 |  |  | 0 | 112/10  8.93% |  |  |  |  |  |  |  |
| 66 | T. Singh | 2022 | India | 208 | | | 292 | | | CS 9300 | |  | 29.95 |  |  |  | 253/48  18.9% |  |  | 247/62  25.1% |  |  |  |  |
| 67 | P. Somasundaram | 2017 | India | 171 | | | | | | KODAK | |  | 28_ 50 |  |  |  |  | 171/16  9.3% |  |  |  |  |  |  |
| 68 | R. K. Yadav | 2023 | India | 316 | | | 364 | | |  | |  | 17_60 |  |  |  | 680/108  15.88% |  |  |  | 316/32 | 364/76 |  |  |
| 69 | R. Arayasantiparb | 2021 | *Thailand* | 292 | | | 142 | | | Morita | | 0.125 |  | 349/13  3.72% | 416/2  0.48% |  |  |  |  |  |  |  |  |  |
| 70 | S. Demirbuga, | 2013 | Turkey | 337 | | | 268 | | | Newtom | |  | 35.7 |  |  | 823/7  0.85% | 925/38  4/1% |  |  |  | LM1  461/6  LM2  516/26 | LM1  362/1  LM2  409/12 |  |  |
| 71 | H. H. Jo | 2016 | Korea | 518 | | | 393 | | | Hitachi | | 0.3 | 21.9 |  |  |  |  |  | 1786/15  0.8% | 1767/48  2.7% |  |  |  |  |
| 72 | H. S. Kim | 2018 | Korea | 159 | | | 111 | | | Willmed | | 0.2 | 27.7 |  |  |  | 540/215  39.8% |  |  |  | 318/152 | 222/63 | nc | nc |
| 73 | S. E. Yang | 2021 | Korea | 591 | | | 663 | | | HDX Corp | | 0.2 | 44.7 |  |  |  | 2508/924  36.8% |  |  |  | 1326/574 | 1182/350 | 1254/455 | 1254/469 |
| 74 | K. Abdalrahman | 2022 | Iraq |  | | | | | | sirona | | 0.125 | LM 2  28.33 |  |  |  | 368/64  17.4% |  |  | 369/29  7.9% | LM2  204/47  UM2  218/20 | LM2  164/17  UM2  151/9 | LM2  185/31  UM2  187/18 | LM2  183/33  UM2  182/11 |
|  |  |  |  |  |  |  |  |  |  |  |  |  | UM2  32.68 |  |  |  |  |  |  |  |  |  |  |  |
| 75 | A. Nouroloyouni | 2023 | Iran |  | | | | | | Newtom | | 0.2 |  |  |  | 248/11  4.4% | 478/20 |  |  |  | nc | nc |  |  |
| 76 | S. Sönmez Kaplan | 2021 | Turkey | 368 | | | | | 306 | Sirona | | 0.25 | 36.42 |  |  |  | 1348/144  10.7% |  |  |  |  |  |  |  |
| 77 | T. Funakoshi | 2021 | Japan | 657 | | | | | | Asahi  Roentgen | | 0.1  0.2 |  |  |  |  | 1058/330  31.2% |  |  |  |  |  |  |  |
| 78 | I. Kaya Büyükbayram | 2019 | Turkey | 100 | | | | | 92 | Morita | | 0.25 | 43.5 | 327/15  4.58% | 264/3  1.13% |  |  |  |  |  |  |  |  |  |
| 79 | M. Mashyakhy | 2019 | Saudi Arabia | 108 | | | | | 100 | Morita | | 0.25 | 26 |  |  |  |  |  | 354/2  0.6% | 372/4  1.1% |  |  | UM1  176/1  UM2  186/4 | UM1  178/1  UM2  186/0 |
| 80 | W. C. Ngeow | 2020 | *Malaysia* | 26 | | | | | 35 | iCAT | | 0.3 | 29.8±9.6 |  |  | 115/5  4.3% | 111/2  1.8% |  |  |  |  |  | LM1  58/3  LM2  54/2 | LM1  57/2  LM2  57/0 |
| 81 | H. Alfawaz | 2019 | Saudi Arabia | 259 | | | | | 228 | Planmeca  carestream | | 0.2  0.3 | 30 |  |  | 529/1  0.19% | 681/62  9.1% |  |  |  | LM2  339/46 | LM2  342/16 |  |  |
| 82 | B. Aricioğlu | 2021 | Turkey | 211 | | | | | 186 | Planmeca | | 0.2 | 16_ 75 | 538/113  21% | 503/29  5.8% | 296/29  9.8% | 461/119  25.8% |  |  |  | LPM1  278/34  LPM2  264/5  LM1  148/16  LM2  245/69 | LPM1  260/69  LPM2  239/24  LM1  148/13  LM2  216/50 |  |  |
| 83 | R. Chaintiou Piorno | 2021 | *Argentina* | 170 | | | | | | Planmeca | | 0.15  0.2 |  | 269/27  10% | 231/4  2% |  |  |  |  |  | nc | nc |  |  |
| 84 | A. J. Alenezi | 2022 | Saudi Arabia | 87 | | | | 62 | | Cybermed | |  | 16_ 65 |  |  |  | 199/26  13.07% |  |  | nc | nc | na | 103/12 | 96/14 |
| 85 | D. S. Abdulateef | 2021 | Iraq | 314 | | | | 249 | | Sirona | | 0.125 |  | 936/15  1.6% | 785/6  0.76% |  |  |  |  |  | LPM1  511/5  LPM2  448/2 | LPM1  425/10  LPM2  337/4 | LPM1  439/9  LPM2  383/3 | LPM1  497/6  LPM2  402/3 |
| 86 | S. Srivastava | 2019 | Saudi Arabia | 74 | | | | 82 | | Sirona | | 0.15  0.3 | 38.5 | 276/48  17.4% | 258/19  7.4& |  |  |  |  |  |  |  |  |  |
| 87 | S. Y. Kim | 2016 | Korea | 960 | | | | | | Willmed | | 0.167 |  |  |  |  | 1920/770  40% |  |  |  | 1038/487 | 882/283 | 960/378 | 960/392 |
| 88 | A. M. Pawar | 2017 | India | 532 | | | | |  | Planmeca | | 0.1 | 26.8 |  |  |  | 983/129  13.12% |  |  |  | 494/76 | 489/53 |  |  |
| 89 | S. Živanovic | 2021 | Serbia | 73 | | | | | 77 | Sirona | | 0.16 | 39 |  |  |  | 199/11 |  |  |  |  |  |  |  |
| 90 | E. Pedemonte | 2018 | Chile |  | | | | | | Morita | | 0.25 |  | 201/18 | 201/1 |  |  |  |  |  |  |  |  |  |
| 91 | D. G. Seo | 2012 | Korea | 108 | | | | | | Genoray | | 0.16 | 25 |  |  |  | 216/92  42.6% |  |  |  |  |  |  |  |
| 92 | S. Suresh | 2023 | Pakistan | 29 | 37 | | | | |  | |  |  |  |  |  | 132/14 |  |  | 132/5 | UM2  58/3  LM2  58/7 | UM2  74/2  LM2  74/7 |  |  |
| 93 | M. Feghali | 2022 | Lebanon | 125 | 132 | | | | | Newtom | | 0.2 | 36.31±14.34 |  |  | 296/3 | 352/32 |  |  |  | nc | nc |  |  |
| 94 | A. Haddadi | 2019 | Iran | 260 | | | | | | sordex | | 0.13 |  |  |  |  | 260/30  11/5% |  |  |  | na | nc |  |  |
| 95 | D. Helvacioglu | 2013 | Turkey | 91 | | | 69 | | | icat | | 0.25 | 39 |  |  |  | 271/24 |  |  |  | nc | nc | nc | nc |
| 96 | I. A. Sherwood | 2019 | India | 417 | | | 341 | | | carestream | | 0.09 | 33.84 |  |  |  | 814/57 |  |  |  |  |  |  |  |
| 97 | Y. C. Wu | 2020 | Taiwan | 155 | | | 225 | | | Newtom | |  | 40.4±16.1 |  |  | 760/2 | 760/340 |  |  |  |  |  |  |  |
| 98 | J. Abarca | 2020 | *Chile* | 289 | | | | | | iCAT | | 0.2 | 30 |  |  |  | 512/56 |  |  |  |  |  | nc | nc |
| 99 | M. Tassoker | 2018 | *Turkey* | 133 | | | 123 | | | Morita | |  | 32.3 |  |  |  | 444/47 |  |  |  |  |  |  |  |
| 100 | Q. Guo | 2023 | china | 150 | | | 134 | | | kavo | | 0.15 | 42.52±3.22 |  |  |  | 568/202 |  |  |  |  |  |  |  |
| 101 | O. Rae | 2023 | Australia | 595 | | | 285 | | | Morita  iCAT | | 0.08  0.2 | 29 | 1576/52 | 1424/14 |  |  |  |  |  |  |  |  |  |

**Table 1.** Details of included studies

F: Female, M: Male, PML1: Lower(Mandibular) First Premolar, PML2: Lower(Mandibular)Second Premolar, LM1: Lower(Mandibular) First Molar, LM2: Lower(Mandibular) Second Molar, LM3: Lower(Mandibular) Third Molar, UM1: Upper(Maxillary) First Molar, UM2: Upper(Maxillary) Second Molar, nc : Information not clearly stated in the studies
