# Supplementary material for: Prevalence of c-shaped canal morphology in premolar and molar teeth assessed by cone-beam computed tomography: systematic review and meta-analysis
Source: BMC Oral Health. 2025 Oct 22;25:1657. doi: 10.1186/s12903-025-06946-8 (PMC12542057; doi:10.1186/s12903-025-06946-8)
Supplement: Supplementary file 2 — Supplementary Material 2. [file 12903_2025_6946_MOESM2_ESM.docx]

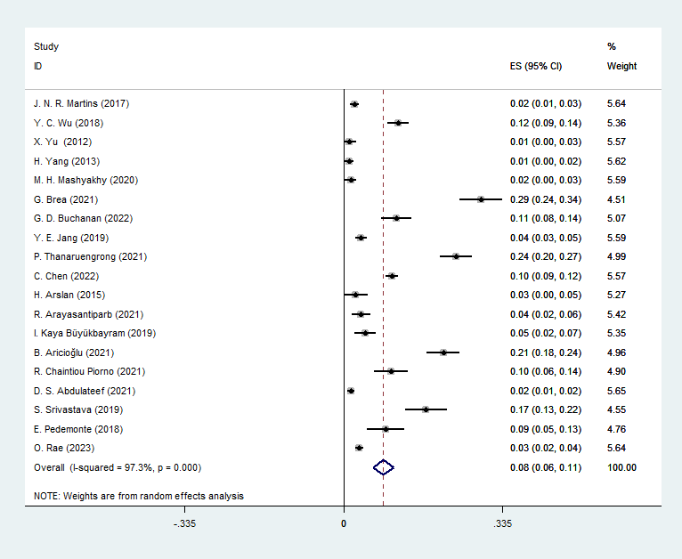


Fig. 1. C-shaped canal prevalence of mandibular first premolar


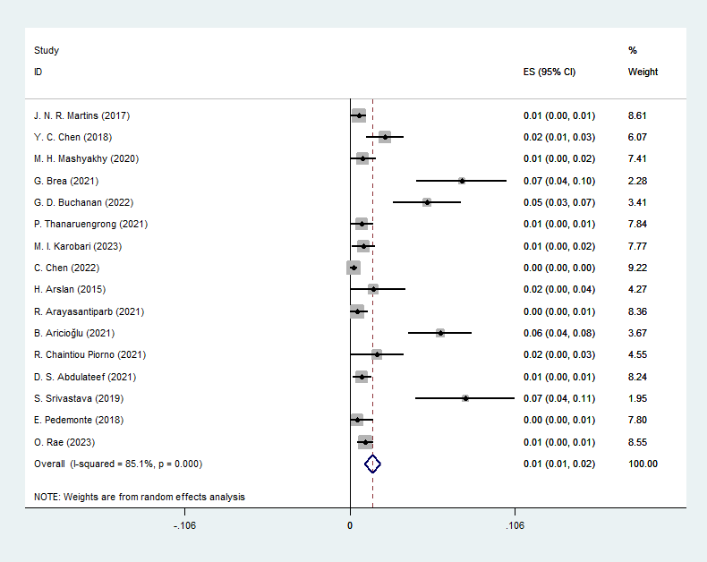


Fig. 2. C-shaped canal prevalence in the mandibular second premolar


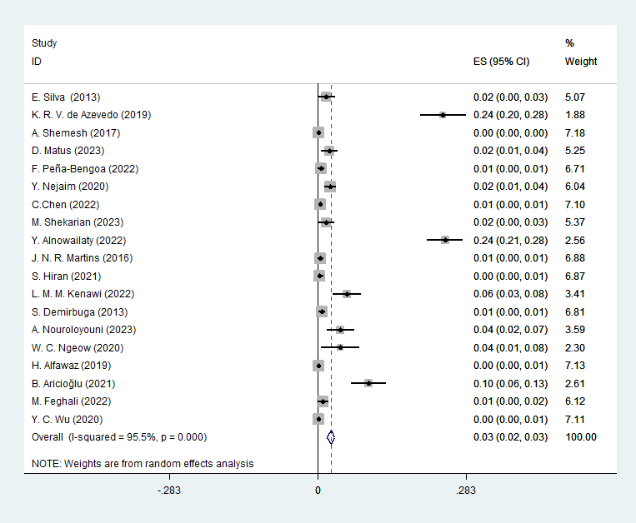


Fig. 3. C-shaped canal prevalence in the mandibular first molar


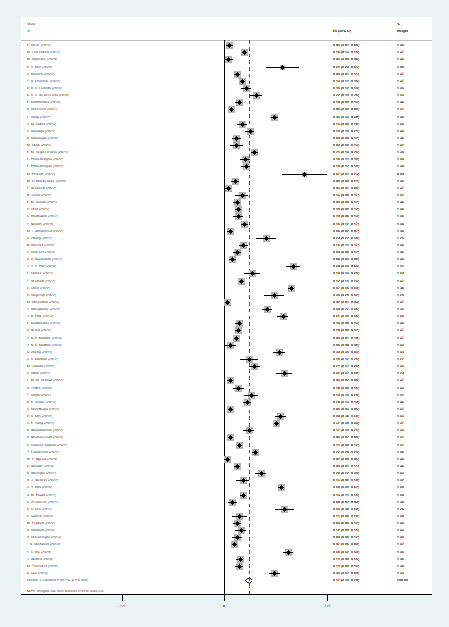


Fig. 4. C-shaped canal prevalence in the mandibular second molar


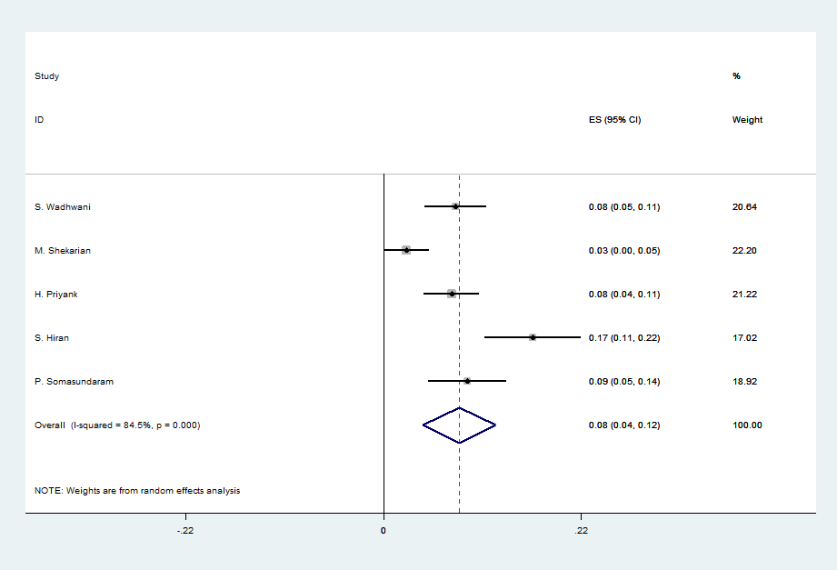


Fig. 5. C-shaped canal prevalence in the mandibular third molar


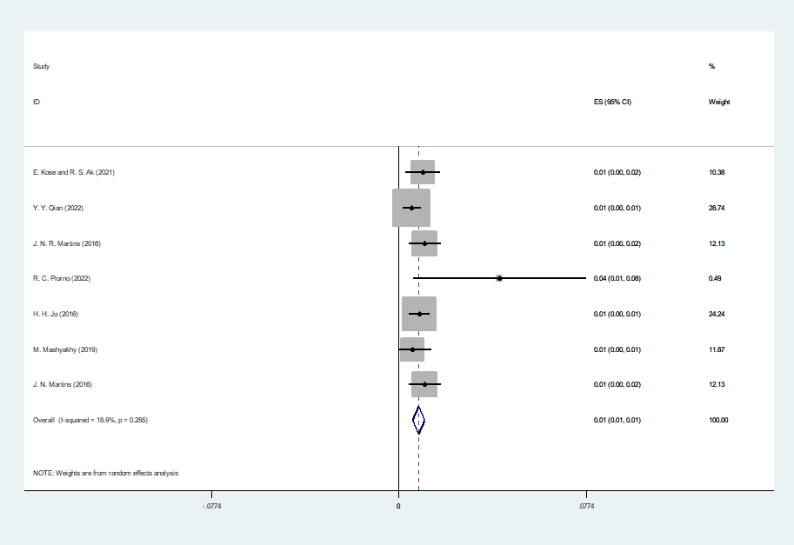


Fig. 6. C-shaped canal prevalence in the maxillary first molar


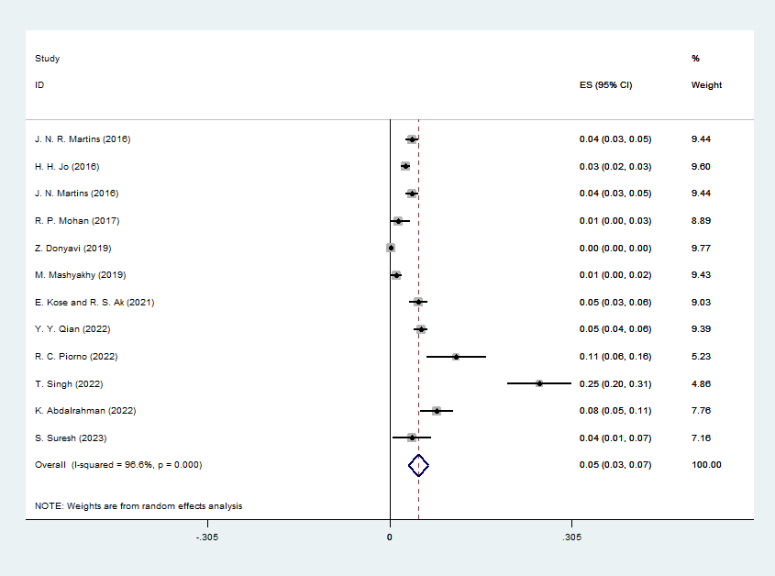


Fig. 7. C-shaped canal prevalence in the maxillary second molar


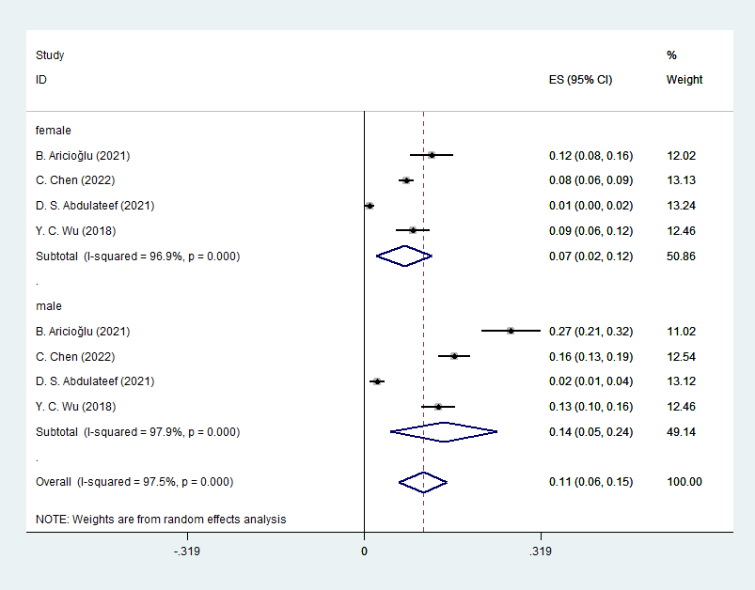


Fig. 8. C-shaped canal prevalence in the mandibular first premolar in female and male


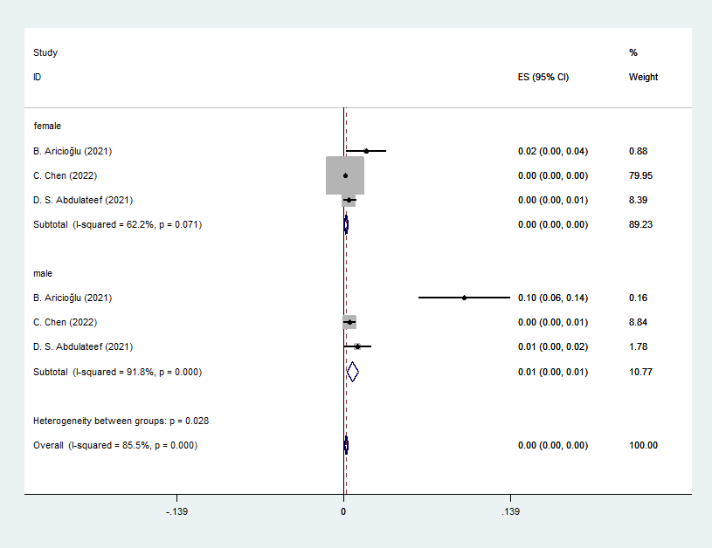


Fig. 9. C-shaped canal prevalence in the mandibular second premolar in female and male


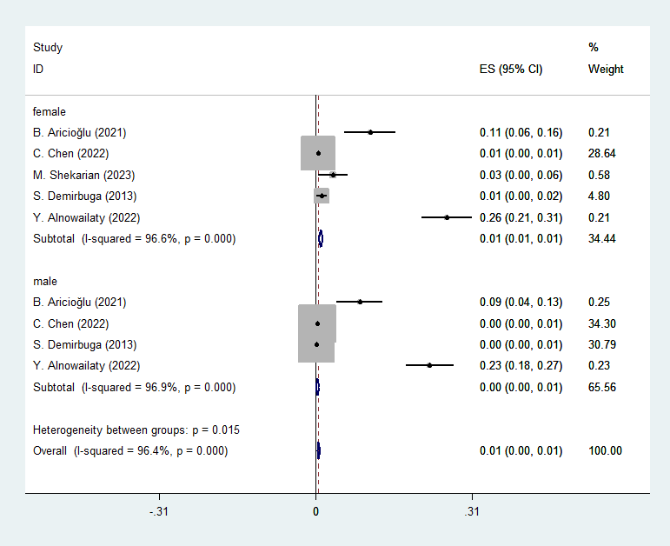


Fig. 10. C-shaped canal prevalence in the mandibular first molar in female and male


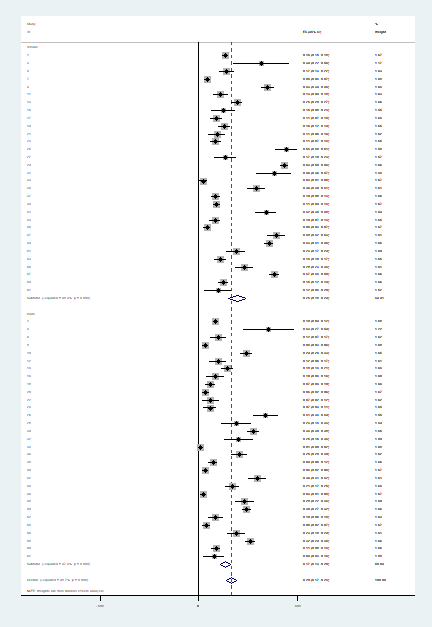


Fig. 11. C-shaped canal prevalence in the mandibular second molar in female and male


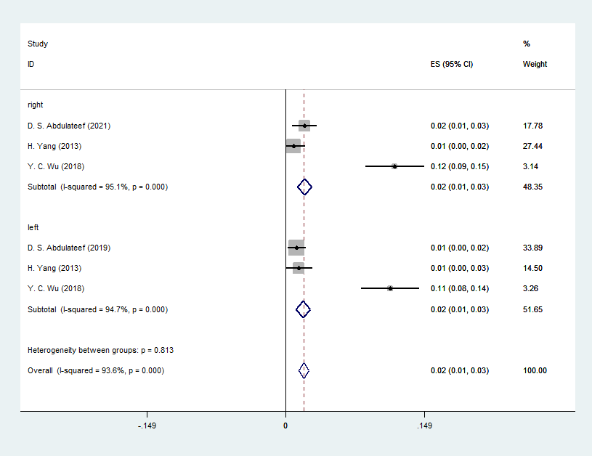


Fig. 12. C-shaped canal prevalence in the mandibular first premolar on the right and left sides


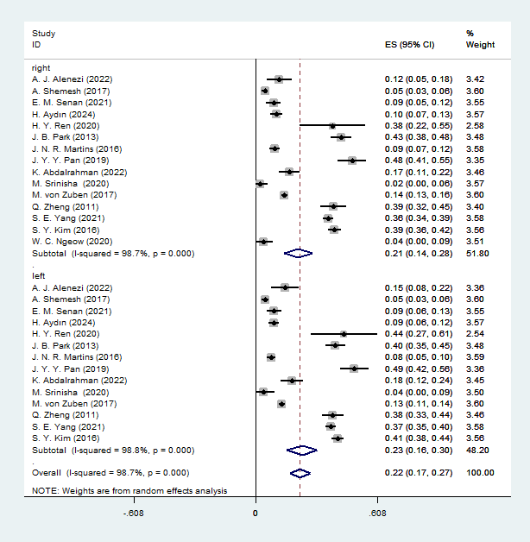


Fig. 13. C-shaped canal prevalence in the mandibular second molar on the right and left sides


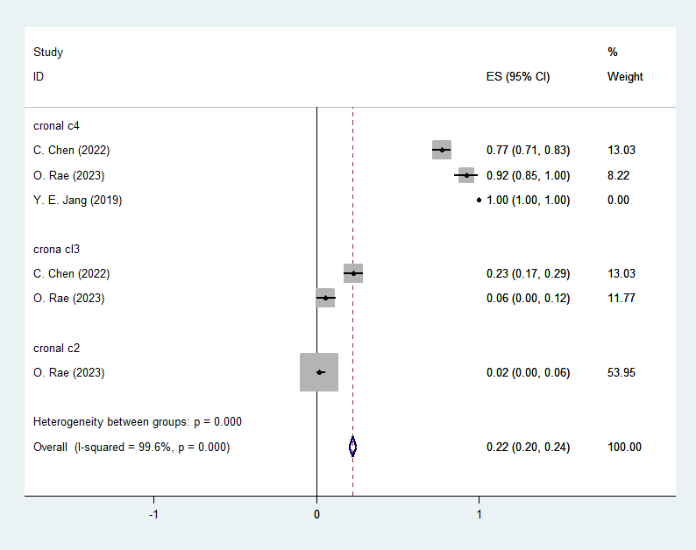


Fig. 14. Classification of C-shaped canals of the mandibular first premolar in the coronal section


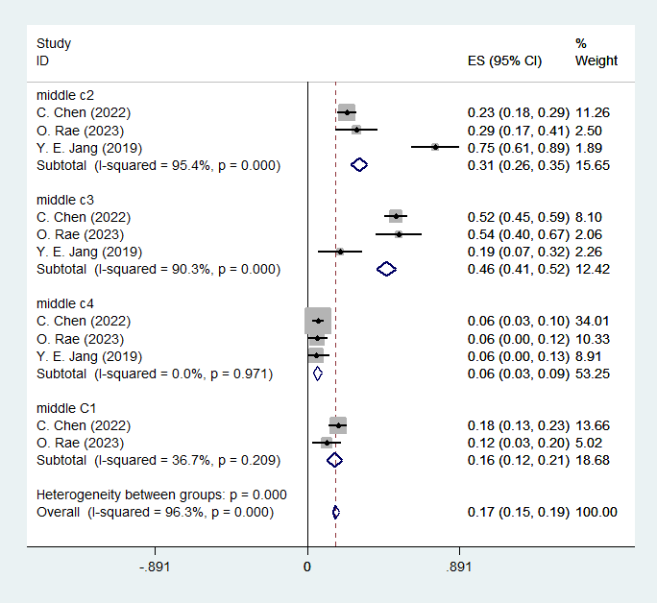


Fig. 15. Classification of C-shaped canals of the mandibular first premolar in the mid-section


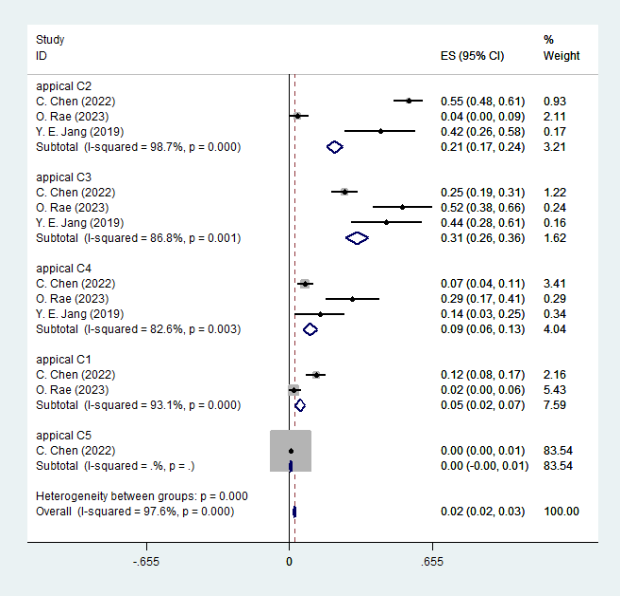


Fig. 16. Classification of C-shaped canals of the mandibular first premolar in the apical section


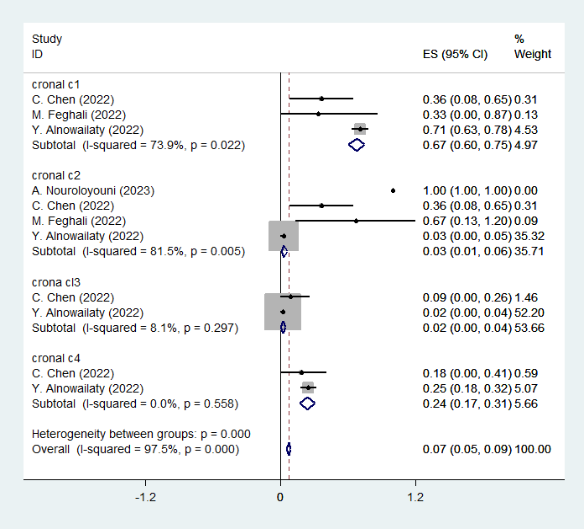


Fig. 17. Classification of C-shaped canals of the mandibular first molar in the coronal section

Fig. 18. Classification of C-shaped canals of the mandibular first molar in the mid-section


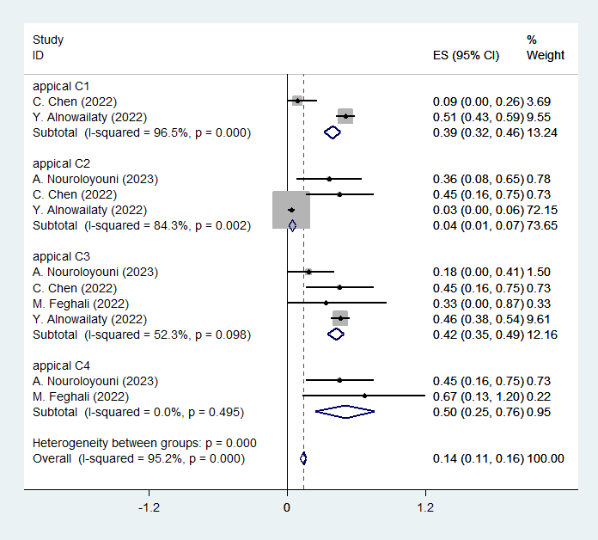


Fig. 19. Classification of C-shaped canals of the mandibular first molar in the apical section


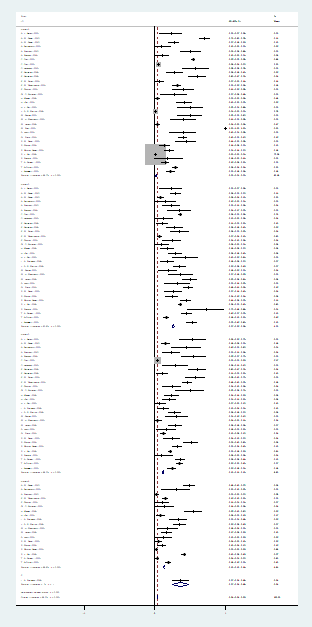


Fig. 20. Classification of C-shaped canals of the mandibular second molar in the coronal section


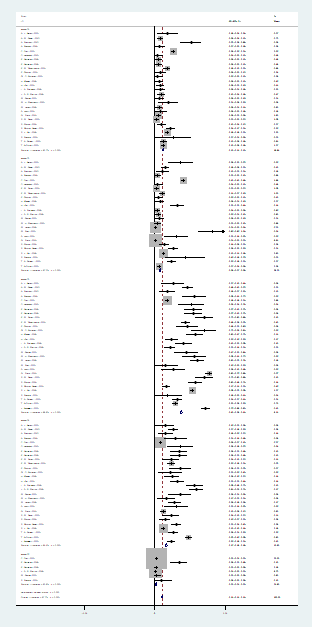


Fig. 21. Classification of C-shaped canals of the mandibular second molar in the mid-section


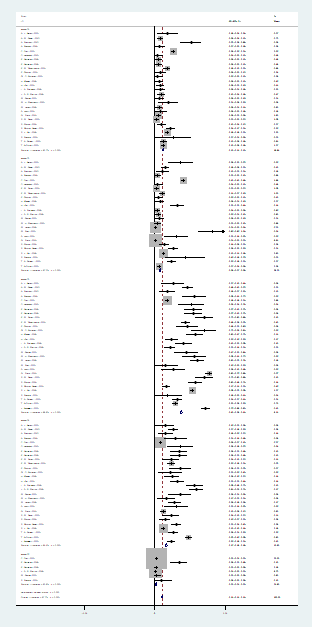


Fig. 22. Classification of C-shaped canals of the mandibular second molar in the apical section


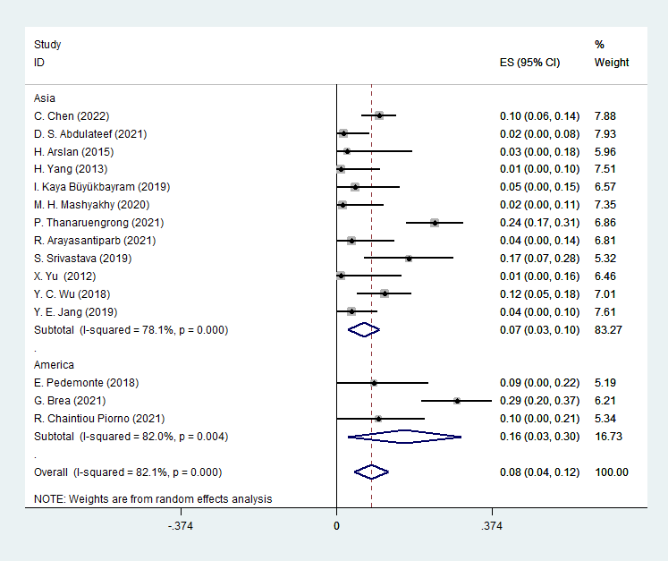


Fig. 23. Distribution of the mandibular first premolar with a C-shaped canal by the continent


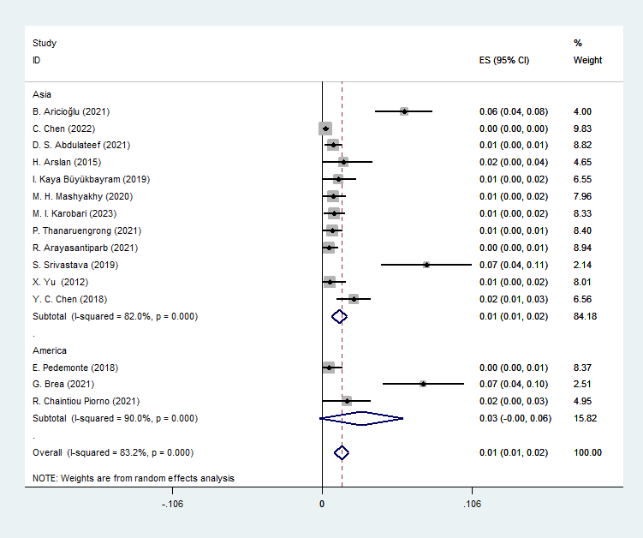


Fig. 24. Distribution of the mandibular second premolar with a C-shaped canal by the continent


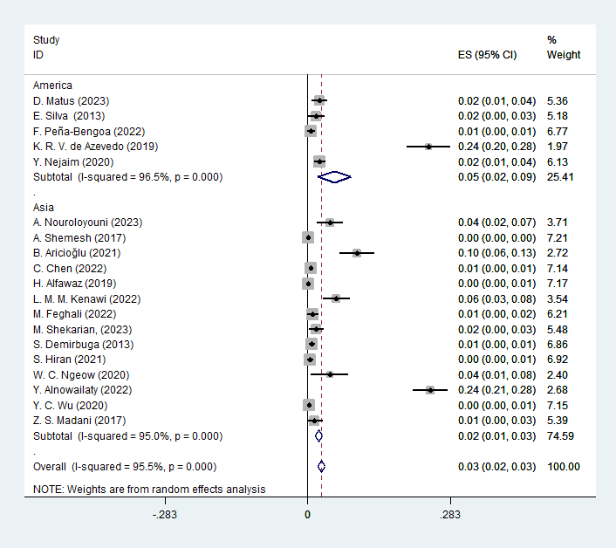


Fig. 25. Distribution of the mandibular first molar with a C-shaped canal by the continent


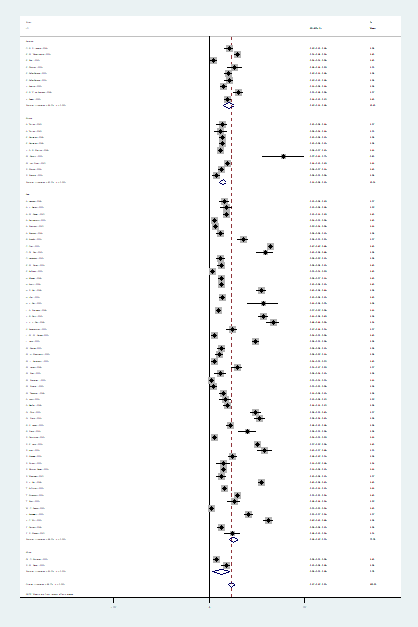


Fig. 26. Distribution of the mandibular second molar with a C-shaped canal by the continent


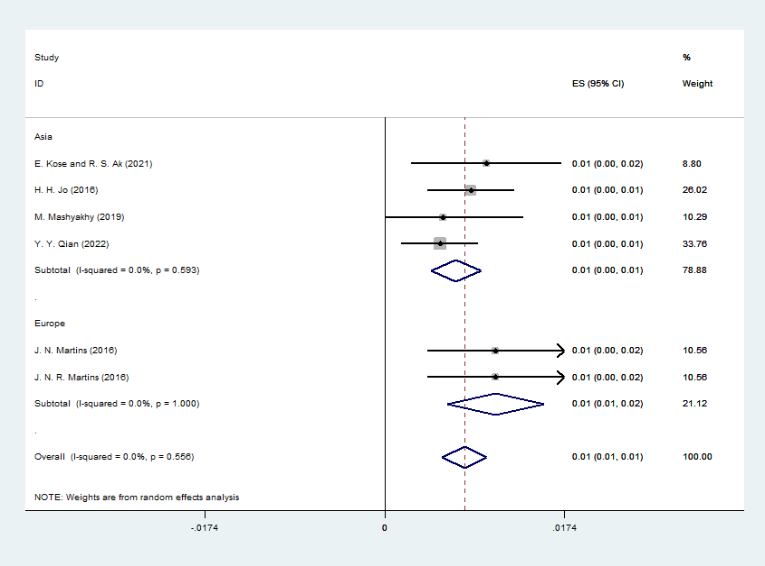


Fig. 27. Distribution of the maxillary first molar with a C-shaped canal by the continent

Fig. 28. Sensitivity analysis for mandibular first premolar


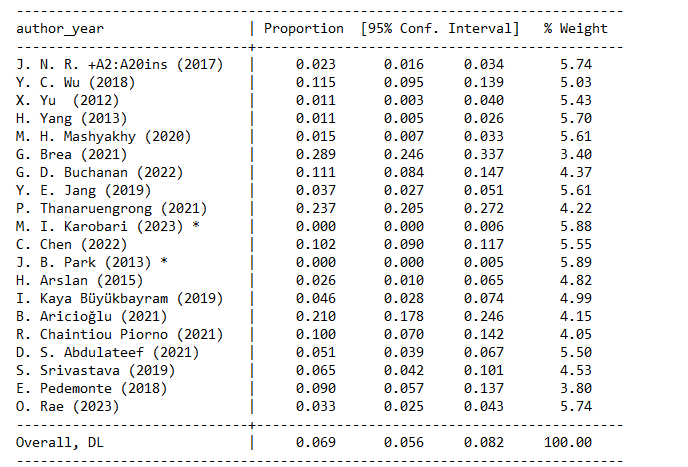


Fig. 29. Sensitivity analysis for mandibular first premolar after exclusion of R. Arayasantiparb (2021)

Fig. 30. Sensitivity analysis for mandibular second premolar


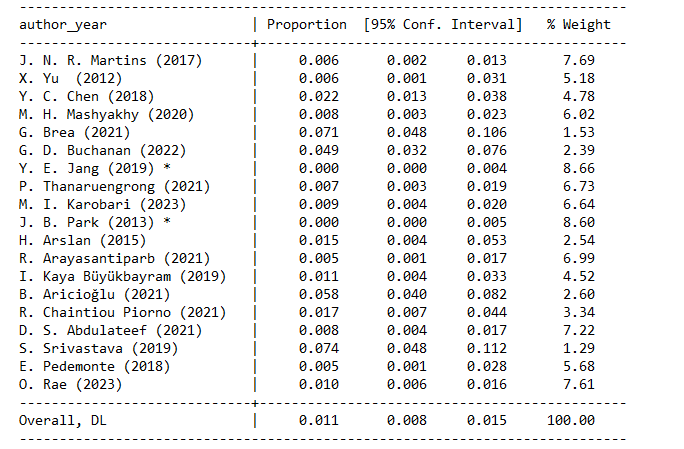


Fig. 31. Sensitivity analysis for mandibular second premolar after exclusion of **C. Chen (2022)**

Fig. 32. Sensitivity analysis for mandibular first molar

Fig. 33. Sensitivity analysis for mandibular second molar

Fig. 34. Sensitivity analysis for mandibular third molar

Fig. 35. Sensitivity analysis for maxillary first molar

Fig. 36. Sensitivity analysis for maxillary second molar


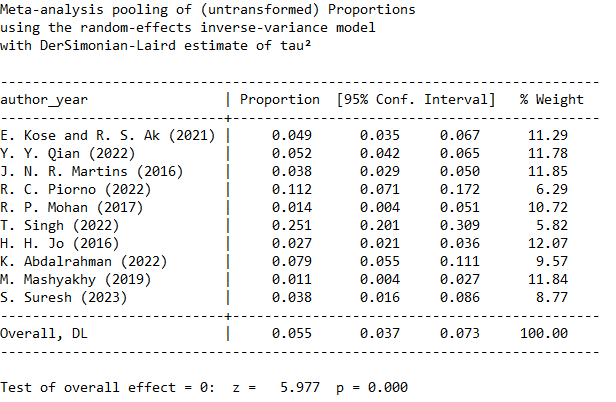


Fig. 37. Sensitivity analysis for maxillary second molar after exclusion of **Z. Donyavi (2019)**
